# Quantum-optimized spatio-temporal transformer for multimodal cardiovascular risk prediction from clinical and wearable data

**DOI:** 10.3389/fcvm.2026.1824194

**Published:** 2026-08-20

**Authors:** S. Sathya, K. Saranya

**Affiliations:** 1 Department of Computer Science and Engineering, Hindusthan College of Engineering and Technology, Coimbatore, Tamil Nadu, India; 2 Department of Computer Science and Engineering, Bannari Amman Institute of Technology, Sathyamangalam, Tamil Nadu, India

**Keywords:** cardiovascular risk prediction, electronic health records, explainable artificial intelligence, feature selection, imbalanced medical datasets, multimodal clinical data, quantum-inspired optimization, spatio-temporal transformer

## Abstract

**Introduction:**

Cardiovascular risk prediction remains a difficult problem due to the extreme class imbalance, time sparsity, non-uniform sampling, and heterogeneity of electronic health record and wearable sensor streams in the real world.

**Methods:**

A multimodal cardiovascular risk prediction framework called Quantum-Optimized Spatio-Temporal Transformer (QST-Trans) was designed. The framework combines Contextual Dual-Stream Synchronization to facilitate alignment between irregular clinical and wearable measurements, Enhanced Quantum-Inspired Binary Grey Wolf Optimization to select features with feature redundancy, a spatio-temporal transformer including attention gating and Kolmogorov-Arnold Network (KAN) layers, and time-aware SHAP to interpret the model's predictions at the patient level based on their longitudinal risk courses. The MIMIC-III and CAIR-CVD-2025 and UCI Heart Disease data sets were used to evaluate.

**Conclusions:**

The proposed QST-Trans framework successfully achieved AUC-ROC 0.99, F1-score 0.98, and sensitivity 97.5%, outperforming the considered baseline methods.

**Discussion:**

The results suggest that multimodal synchronization, quantum-inspired feature optimization, spatio-temporal learning and the inclusion of time-aware explainability are a promising approach to cardiovascular risk stratification. But a multi-center validation and clinical testing in the real world should be required before clinical adoption.

## 1 Introduction

Cardiovascular diseases (CVDs) remain the major cause of death in the global arena, contributing a significant percentage of morbidity and medical spending (1). Regardless of the progress in clinical diagnostics and preventive care, it is still difficult to identify people with high cardiovascular risk in time because the disease is multifactorial and progressive (2). Rapid and dependable risk forecasting is thus crucial in facilitating preventive measures, streamlining clinical judgments and minimizing unwarranted poor outcomes (3).

The latest developments in the medical data acquisition have facilitated the acquisition of heterogeneous multimodal data, such as electronic health records, intensive care unit (ICU) signals, and portable sensors continuous streams (4). Although these sources of data provide unprecedented possibilities of the individual cardiovascular risk evaluation, they pose significant technical notions. Clinical data are associated with high modality heterogeneity, extreme temporal sparsity across sources, and strong imbalance in classes with a huge number of healthy samples in comparison to at-risk cases (5, 6). The presence of irregular sampling periods, gaps, and shifts in distribution between wearable and clinical streams of multimodal cardiovascular data, point further to the necessity of tremendous synchronization and imbalance-sensitive modeling techniques using the exploratory analysis.

Traditional machine learning models mainly use the form of stationary tabular features and cannot express temporal dynamics and long-range relationships of cardiovascular progression (7). Sequential deep learning models like long short-term memory (LSTM) networks partially tackle the task of temporal models but have difficulties with irregular sampling, sparse time series, and missing data are typical of clinical scenarios in practice (8). More recently, transformer-based architectures have proven better ability to capture long range temporal relations; but current transformer implementations have high computational cost, inefficient use of features, and low interpretability, most of which can be used in safety-critical medical applications (9–11).

To overcome the challenges, the current paper proposes a Quantum-Optimized Spatio-Temporal Transformer (QST-Trans) framework of multimodal cardiovascular risk prediction. The main findings by this work are as follows:

1. Contextual Dual-Stream Synchronization (CDSS) system to synchronize irregularly sampled clinical data with continuous wearable signals.
2. An improved Quantum-Based Binary Grey Wolf Optimization (EQI-BGWO) model of redundancy-sensitive and stability-driven selection of features using heterogeneous clinical features.
3. A spatio-temporal transformer architecture with attention gating and KolmogorovArnold Network (KAN) layers to effectively model complex temporal and cross-modal interactions.
4. An interpretability module of SHAP with time awareness (T-SHAP), which allows explaining risk trajectories in patients and providing clinically interpretable decision support.
5. Wholesome validation on a variety of benchmark cardiovascular data with high predictive power, as well as strong performance with class imbalance and better clinical interpretability over state-of-the-art baselines.

The rest of the paper is organized in the following way. Part II gives a review of the pertinent literature in cardiovascular disease prediction, including conventional machine learning and deep learning models, time-series clinical modeling methods, feature selection and optimization methods, transformer-based models in health care, and recent innovations in explainable artificial intelligence in medical decision support. Section 3 covers the exploratory data analysis, which consists of a description of the datasets and important visual insights that justify the modeling decisions. Section 4 explains the proposed methodology, which consists of the general architecture of the system, data preprocessing, and contextual dual-stream synchronization, quantum-inspired EQI-BGWO feature selection, the spatio-temporal transformer framework (QST-Trans), and the time-aware explainability module, based on T-SHAP. Section 5 describes the experiment design, such as dataset divisions, control models, hyperparameters, and performance indicators. Section 6 presents the experimental results and analysis, including the results of quantitative performance comparison, training dynamics, evidence in form of visualization, and interpretability. Section 7 speaks about the weaknesses of the proposed approach and gives directions to the further research. Lastly, Section 8 summarizes the paper by highlighting the main contribution and clinical implications of the proposed framework.

## 2 Related work

The recent developments in the field of artificial intelligence and data-driven healthcare analytics have enhanced the task of cardiovascular disease prediction considerably, as machine learning, deep learning, and multimodal data integration methods are used to enhance the process. Initial research mainly investigated the application of traditional machine learning models to fixed clinical characteristics; although the availability of longitudinal electronic health records, wearable sensors streams, and physiological high-frequency measurements has moved research toward more elaborate temporal and multimodal modeling schemes. Modern work has developed deep learning models, such as recurrent neural networks, attention-based models and transformer-based models, to learn complicated time interactions, non-linear feature interactions that are specific to cardiovascular progression. Similar studies have put forward the significance of feature selection and optimization to reduce redundancy, imbalance in classes, and improve model generalization. In addition, explainable artificial intelligence has become a challenging clinical adoption requirement, which allows transparency, trust, and interpretability of predictive results. Through this part, the recent literature on heart disease prediction with the use of ML and DL models, time-series clinical modeling, feature selection and optimization methods, transformer-based healthcare architectures, and explainable AI approaches are discussed in terms of their strengths, limitations, and unsolved issues that drive the proposed QST-Trans framework.

### 2.1 Heart-disease prediction (ML & DL)

Machine learning (ML) and deep learning (DL) prediction of heart diseases have become the topic of great interest because of the growing accessibility of planned clinical data and the improvement of predictive modelling. Random Forest, Support Vector Machine and XGBoost are examples of traditional ML classifier that have been shown to be effective in binary cardiovascular risk classification (12, 13). To make the predictive process even more efficient, convolutional neural networks (CNNs), long short-term memory (LSTM) networks, and ensemble approaches have been suggested to be used as hybrid or deep learning models, which allow to capture the non-linear interactions of features and limited temporal patterns (14, 15). Additional more recent research has investigated self-supervised learning and deep feature representations to enhance generalization especially when using medium sized cardiovascular data (16).

Limitations summary: Although they are effective, most of both ML and DL methods are based on the information of fixed or poorly dynamic representations and are not capable of modeling irregular disease development in heterogeneous sources of information over time. Such restrictions drive the desire to have an integrated spatio-temporal model like QST-Trans, which would be able to forecast multivariate temporal relationships and combine multimodal clinical and wearable data.

### 2.2 Time-series clinical modelling

The time-series modelling is important in dynamic physiological patterns inherent in clinical and wearable health data. RNNs and LSTM networks have been widely used in sequential health surveillance tasks such as ICU surveillance and continuous vital signs monitoring (17). More recent works have moved in this direction towards multimodal and time-aware architectures, which explicitly deal with irregular sampling and missing values with dynamic alignment and fusion processes (18). These frameworks are shown to be more robust than fixed-length temporal modeling, especially in clinical settings, where there is a lack of data (19).

Limitations summary: Time-series models can better represent time but recurrent architectures are poor in capturing long-range dependencies, irregular sample rates and multimodal stream scalability. These difficulties point at the necessity of spatio-temporal modeling using transformers with an explicit mechanism of synchronization, which is achieved in the framework proposed under the name QST-Trans.

### 2.3 Feature selection & optimization

The correct choice and optimization of features is very essential in enhanced cardiovascular risk prediction by eliminating dimensionality, reducing redundancy and improving the generalization. Most popular feature relevance estimation methods based on classical statistics like mutual information, ANOVA, and chi-square tests and evolutionary and heuristic optimization through evolutionary and heuristic methods have been explored (20). Empirical evidence shows that a properly selected set of feature to classify a set of data can enhance the accuracy of classification to a considerable degree at a lower computational cost and in a more interpretable model (21). Different predictive performances on complicated clinical data have been further optimized using hybrid strategies involving feature selection with ensemble or deep learning pipelines.

Limitations summary: The current method of feature selection is not usually coupled with temporal modelling and is not globally optimized in high dimensional, multimodal environments. This drawback is the driving force behind the incorporation of quantum-inspired feature optimization as part of QST-Trans to allow efficient and redundancy-conscious feature selection before spatio-temporal learning.

### 2.4 Transformers in healthcare

Transformer-based and attention-based architectures have become more prevalent in healthcare analytics in recent times as they can capture long-range dependencies without being limited by recurrent structures. Hybrid transformer models like CardioTabNet and phenotype-aware architectures like ASCENDgpt have been applied to clinical and tabular medical data and shown to be competitive in cardiovascular risk prediction tasks (22, 23). These models exploit self-attention mechanisms to acquire interactions between contextual features and have demonstrated effectiveness both with static and longitudinal healthcare datasets (24).

Limitations summary: Despite their advantages, the current healthcare transformers can be characterized by the lack of clear temporal synchronization, efficiency, and clinical interpretability and can be applied to real-world multimodal settings only to a limited degree. These weaknesses are what drive the creation of QST-Trans that merges synchronized spatio-temporal attention and optimized feature selection to enhance robustness and scalability.

### 2.5 Explainable AI in medicine

Explainable artificial intelligence (XAI) is now necessary to achieve reliable clinical decision support, especially cardiovascular risk prediction, where the clarity of reasoning is paramount to acceptance by clinicians. SHAP and LIME are model-agnostic approaches that have traditionally been combined with ensemble and deep learning models to offer feature-level explanations and enhance interpretability (25). In the recent models, such as XAI-HD, there is an effort to find a balance between predictive performance and transparency, incorporating explainability as a fundamental part of the modeling pipeline, and improve clinician-model interaction (26). Recent studies have further investigated advanced machine-learning classifiers, CNN-based deep learning, comparative cardiovascular prediction frameworks, and feature-selection and generative approaches for improving cardiovascular risk prediction (27–30).

Limitations summary: The majority of current XAI methods give one-time or overall explanations and do not reflect the dynamics of risks in longitudinal patient data. The given limitation spurs the integration of time-conscious explainability (T-SHAP) into QST-Trans, which allows interpreting patient-level risk trajectories dynamically and in accordance with clinical reasoning.

A comparative analysis of the representative state-of-the-art cardiovascular risk prediction methods with the proposed QST-Trans framework is provided in Table 1, with the differences in data modality processing, time model, feature optimization, and explainability.

**Table 1:** Comparative analysis of existing works and the proposed QST-trans framework.

Ref.	Model/Approach	Data Type	Temporal Modeling	Feature Selection	Explainability	Key Limitation
(12)	Ensemble DL + FS (Al-Mahdi et al.)	Clinical tabular	✗	✔ (heuristic)	✗	Ignores temporal dynamics
(13)	Hybrid Ensemble + SHAP	Clinical tabular	✗	✗	✔	Static risk estimation
(14)	Wearable DL (CNN/LSTM)	Wearable signals	✔	✗	✗	Limited multimodal fusion
(15)	Residual GRU + MHSA	Tabular	Partial	✗	✗	Sensitive to irregular sampling
(16)	SimCLR–SHAP	Clinical tabular	✗	✗	✔	No temporal attribution
(17)	RNN/LSTM Survey	Time-series	✔	✗	✗	Limited interpretability
(18)	MedM2T	EHR + ECG	✔	✗	Partial	No feature optimization
(19)	DT-ICU	Multimodal ICU	✔	✗	Partial	High computational cost
(20)	MI/ANOVA *χ*^2^ FS	Clinical tabular	✗	✔	✗	No deep temporal modeling
(21)	Bee Algorithm FS	Clinical tabular	✗	✔	✗	Handcrafted feature bias
(22)	CardioTabNet	Tabular Transformer	✗	✗	✗	No temporal awareness
(23)	ASCENDgpt	EHR	✔	✗	✗	Limited explainability
Proposed	QST-Trans	Clinical + Wearable	✔✔	✔ (EQI-BGWO)	✔ (T-SHAP)	—

### 2.6 Technical insights and comparative discussion

The concept of the QST-Trans framework is effective in overcoming the main shortcomings of the current model of cardiovascular risks prediction by integrating multimodal temporal modelling, feature efficiency, and interpretability. Conventional machine learning and ensemble approaches do well with unchanging data sets but cannot model the mechanisms of disease dynamics and cross-modal interactions, whereas recurrent neural networks like LSTM and GRU cannot deal with irregular sampling, missing data, and long-range dependencies that are found in clinical and wearable data in the real world. Even though transformer-based models enhance the modelling of long-term dependencies, most healthcare applications do not have explicit features optimization and interpretability, resulting in unnecessary focus on attention and high computational costs. The solutions to these problems in QST-Trans include Contextual Dual-Stream Synchronization of contextual temporal alignment, quantum-inspired EQI-BGWO feature selection of redundancy removal and stable convergence, and time-aware SHAP (T-SHAP) of longitudinal risk attribution. In general, the framework provides better predictive strength, clinical explainability, and scalable cardiovascular risk stratification that can be applicable in clinical decision support in the real world.

### 2.7 Research gap and motivation

Current cardiovascular prediction systems tend to have issues with multimodal heterogeneity, irregular time sampling, and limited interpretability. Conventional methods of machine learning use fixed representations and recurrent models are vulnerable to missing data and long-range interactions. Despite the fact that transformer architectures can be used to better temporal model, most current healthcare transformers do not explicitly optimize features and provide clinically interpretable reasoning. The QST-Trans framework proposed will incorporate contextual multimodal synchronization, quantum-inspired feature optimization, spatio-temporal attention learning and time-aware explainability into a single cardiovascular risk prediction pipeline.

## 3 Exploratory data analysis

In the present study, EDA is also carried out to conduct a systematic analysis of the statistical features, structure, and inherent issues of the multimodal cardiovascular data sets. Since clinical records and wearable sensor streams are heterogeneous, EDA will be an important step to support preprocessing plans, feature optimization, and architecture design choices that will be made in the proposed framework. Data distribution, temporal sparsity, feature redundancy, and class imbalance are the primary aspects of analysis that affect the model robustness and clinical reliability directly.

### 3.1 Dataset description

- MIMIC-III Dataset: Comprises of high-resolution ICU clinical data, such as time-stamped vital sign, laboratory measurements, and demographic attributes, with very irregular sampling rates and large missing values, which are observed in practice in clinical care.
- CAIR-CVD-2025 Dataset: Includes organized clinical, biochemical and lifestyle data gathered using wearable devices and periodic health evaluations, which provides more frequent temporal sampling, but encounters significant demographic and physiological shifts in distribution relative to datasets acquired in hospitals.
- UCI Heart Disease Dataset: A standardized table benchmark dataset of similar clinical characteristics and less missing values, but of limited longitudinal depth and multimodal representation.

The three datasets were not directly combined into a single raw data set as all three are from different clinical settings and patient populations. Rather, each dataset was preprocessed separately with the same preprocessing steps, in particular, by imputing missing values, verifying for duplicate data, normalizing the features, aligning them in time (when applicable), and balancing the classes. After preprocessing, only the clinically common cardiovascular features common across datasets were retained (e.g., demographic features, blood pressure measurements, cholesterol-related features, heart rate, etc. and other features compatible across the datasets) for the creation of a common multimodal feature space. Joint representation was not created for dataset-specific variables that could not be consistently represented across the datasets. This approach guarantees that the statistics of each dataset are maintained while providing a consistent learning process over different multimodal data sources.

It is important to note that the three sets of data have different raw feature sets. For both data sets, there are variables of clinical relevance, as well as variables present in the data, but specific to the context of the data being acquired. Thus, harmonisation of features was performed by locating clinically equivalent variables that are present in all the data sets and mapping them to a common feature schema. For cross-dataset experiments, variables with different clinical meaning were not used so as to maintain consistent model inputs.

### 3.2 EDA figures

#### 3.2.1 Class imbalance analysis

**Figure 1:**
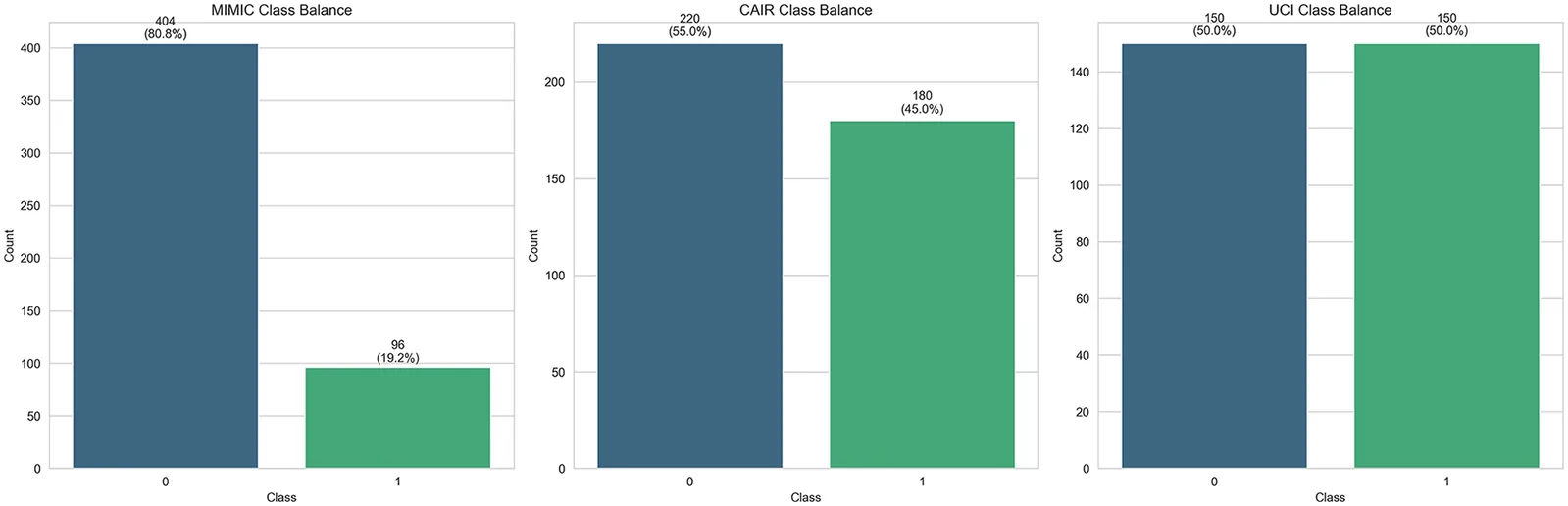
Class imbalance analysis of MIMIC-III, CAIR-CVD, and UCI heart datasets. (Distribution of cardiovascular risk classes across the three datasets, highlighting differences in disease prevalence and class skew).

The analysis of class distribution shows a strong imbalance in all datasets with a strong skew towards non-disease or low risks samples, especially in clinical and population-scale cohorts. MIMIC-III data has a severe imbalance, with 80.8% of the samples being healthy compared to 19.2% being diseased and CAIR-CVD data has moderate imbalance (55.0% healthy vs. 45.0% disease) (refer to Figure 1). On the contrary, the UCI Heart Disease data is balanced and both classes are represented equally. Such comparison suggests that standard training approaches might work with balanced datasets, like UCI, but are likely to face majority-class bias in highly skewed datasets, like MIMIC-III and CAIR-CVD and lead to misleadingly high accuracy and poor disease detection. These results are the direct motivation of the implementation of SMOTE-based class balancing is to optimize minority-class learning and assure the credible cardiovascular risk prediction.

#### 3.2.2 Feature distribution analysis

**Figure 2:**
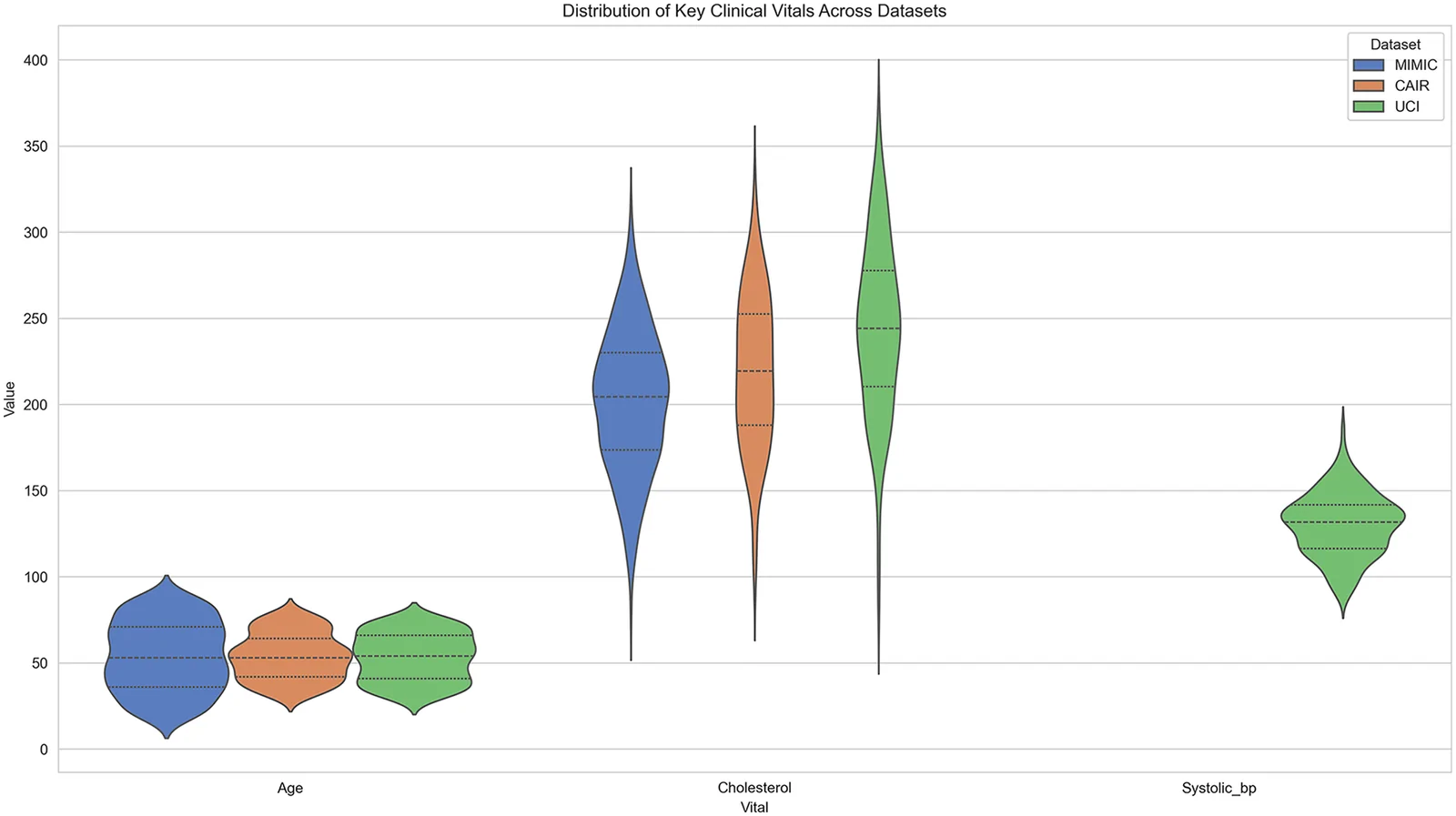
Distribution of key clinical vitals across multimodal cardiovascular datasets (violin plots illustrating dataset-specific variability in age, systolic blood pressure, and cholesterol levels).

The violin plot analysis of the major clinical characteristics, such as age, systolic blood pressure, and cholesterol levels, indicates that there are significant distributional differences in MIMIC-III, CAIR-CVD, and UCI Heart datasets. Compared to CAIR-CVD and UCI cohorts, the number of MIMIC-III patients is older, as there are few patients aged over 80 years, and most of the age distribution is between 50 and 80 years, which is also characterized by a significant demographic change, as shown in Figure 2. In MIMIC-III, cholesterol levels have large ranges (150 300 mg/dL) with heavy tails, indicating that there is acute clinical variability, whereas CAIR-CVD has moderately heterogeneous distributions and UCI has relatively compact ranges. Likewise, the systolic blood pressures are centered on 120,140 mmHg in UCI and extreme outliers are observed in MIMIC-III with a value greater than 180 mmHg, which is typical of emergency and critical care measurements. These distributional discrepancies and heavy-tailed distributions make it clear that covariate shift and model learning can be biased by the presence of outliers that regulate the design choice is to use contextual dual-stream synchronization and normalization to align heterogeneous feature distributions before the spatio-temporal modeling.

#### 3.2.3 Correlation analysis

**Figure 3:**
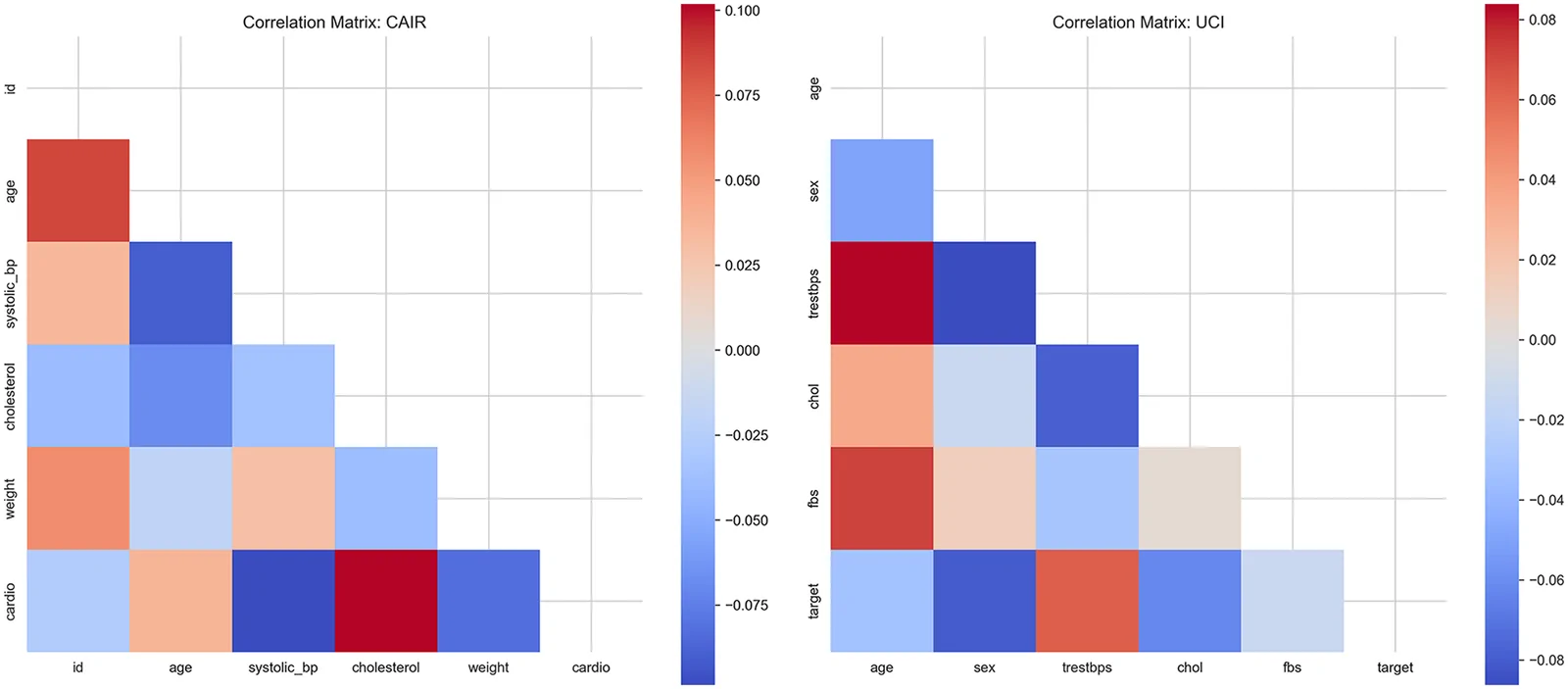
Feature correlation analysis across cardiovascular datasets (correlation heatmaps illustrating inter-feature relationships and redundancy patterns).

The analysis of correlation heatmap shows that several clinical features have the common pairwise correlations across the CAIR-CVD and UCI Heart Disease data sets, which means that feature redundancy and multicollinearity exist. There are moderate positive correlations (e.g., between cholesterol and cardio status or age and systolic blood pressure) and negative correlations between the features, which imply that the content of information is overlapping as shown in Figure 3. The magnitude of individual correlation is small but the recurring nature of the correlated groups of features adds complexity to the model without necessarily proportional performance improvement. Simultaneously, features that are more closely related to the target variable will have a higher likelihood of being useful biomarkers of the cardiovascular system. These results are the direct motivation behind the EQI-BGWO-based feature selection is to eliminate the redundancy of attributes with the remaining clinical features being informative enough to support an easy spatio-temporal model.

#### 3.2.4 Missingness and temporal sparsity analysis

**Figure 4:**
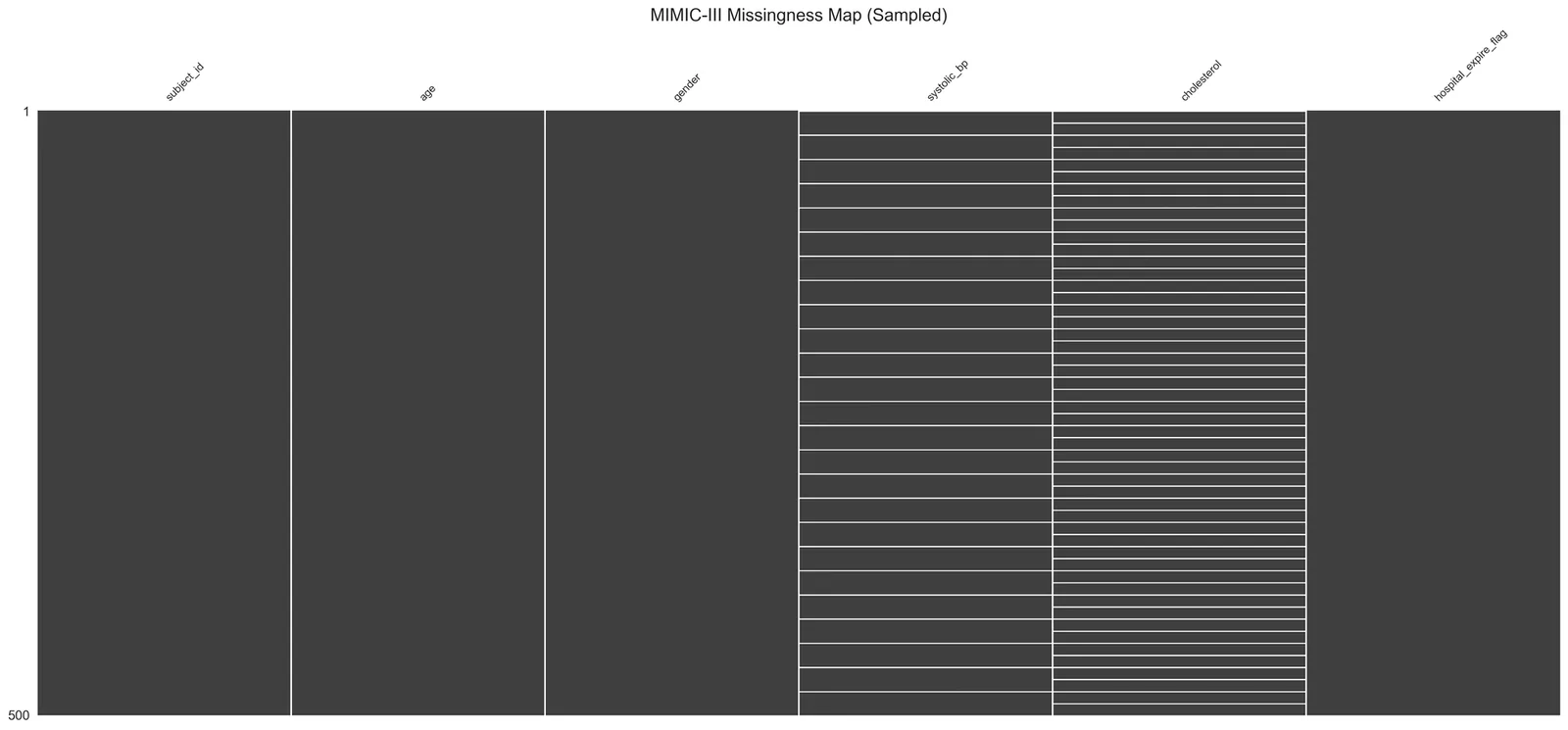
Missingness and temporal sparsity in the MIMIC-III clinical dataset (visualization of feature-level missing data proportions and irregular temporal sampling).

The missingness figure illustrates that the MIMIC-III time-series data has a significant amount of temporal sparsity, especially on ICU-related physiological and laboratory measurements, which are gathered on an as-need basis. As Figure 4 reveals, demographic features, including age and gender, are mostly complete but the main clinical variables, including systolic blood pressure and cholesterol, are missing many important values with the gap percentage of over 4,060 of the total recorded time steps. This trend indicates that the process of collecting data in ICUs is event-based, not constant. High rates of missingness of this magnitude challenge traditional models which make assumptions of dense and regularly sampled inputs. Therefore, this fact inspires the application of a transformer-based architecture with Contextual Dual-Stream Synchronization (CDSS) when modeling irregular time intervals and incomplete multimodal observations is necessary.

#### 3.2.5 Summary of EDA insights

The exploratory analysis demonstrates a strong imbalance between the classes of clinical datasets, especially in MIMIC-III, which requires an imbalance-conscience learning approach. The strong distributional differences in the main physiological variables in the datasets are indicative of cross-domain heterogeneity and the importance of strong normalization and synchronization. Correlation analysis is used to determine clustering of redundant features, which explains the need to perform explicit feature selection to enhance efficiency and generalization. Patterns of missingness reveal extreme temporal sparsity of ICU data that complicates the modeling of dense inputs. All these insights, together, drive the combined design of QST-Trans, which involves the synchronization, optimal feature selection, and spatio-temporal transformer modeling.

## 4 Methodology

**Figure 5:**
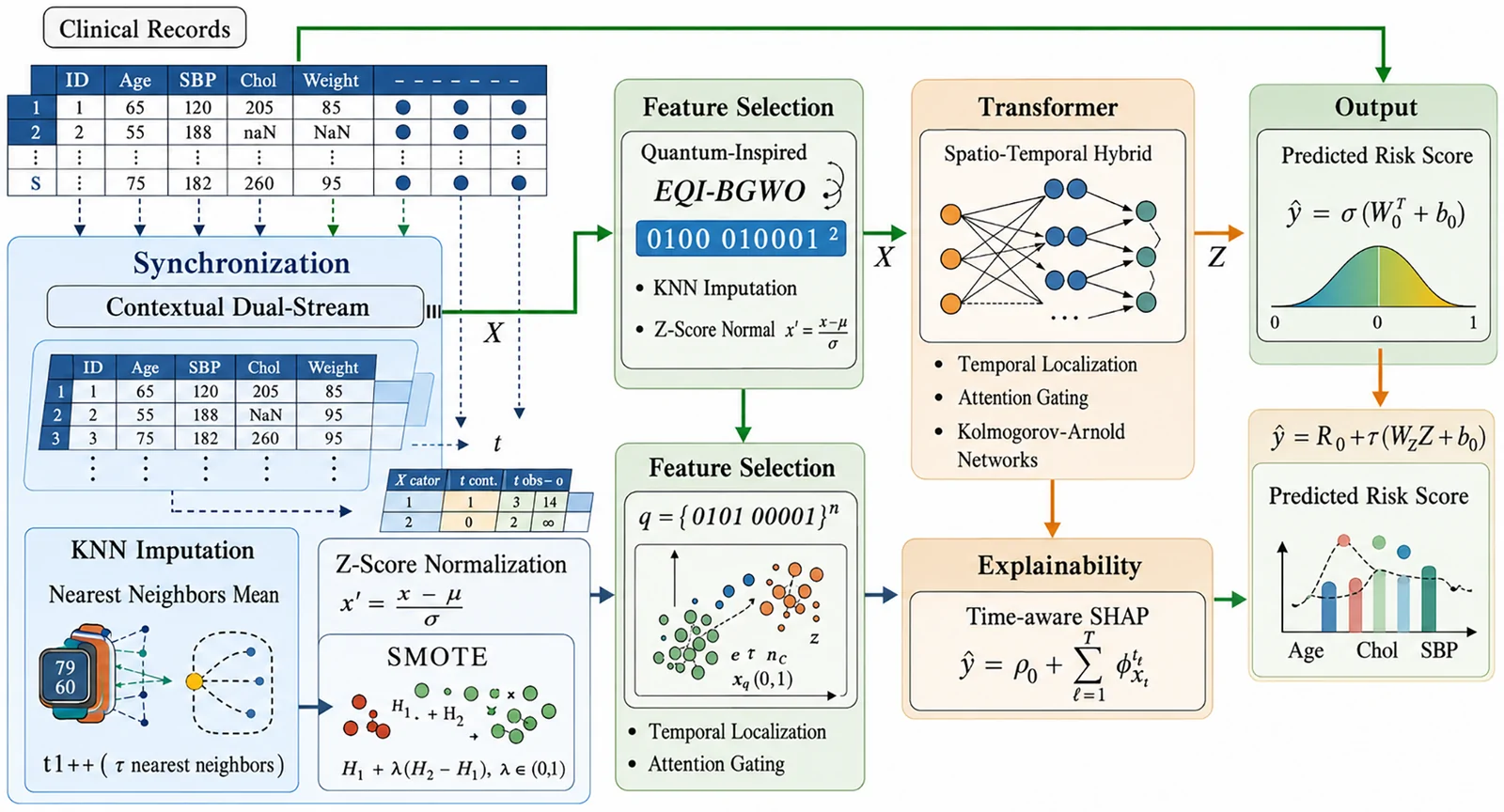
Proposed overall architecture of proposed QST-trans framework. The contextual.

Figure 5 illustrates the overall architecture of the proposed QST-Trans framework for multimodal cardiovascular risk prediction. It uses a heterogeneous input of clinical records and wearable sensor streams which are initially aligned with the Contextual Dual-Stream Synchronization (CDSS) to resolve the irregularity in time and data-sparsity. The raw data are preprocessed to run quantum-inspired EQI-BGWO feature selection in order to come up with a subset of the informative clinical attributes that is optimal and stable. The chosen features will then be put through a spatio-temporal transformer that includes temporal encoding, attention gating, and KolmogorovArnold Network (KAN) layers to capture the compounding temporal interactions and non-linear interactions. Lastly, the framework generates patient level cardiovascular risk scores, with an appendix time-sensitive SHAP (T-SHAP) module, which generates interpretable risk curve information, allowing transparent and clinically significant decision support.

Dual-Stream Synchronization (CDSS) module synchronizes clinical records and wearable sensor streams. They are then synchronized and passed to the feature-selection module, which processes them before being encoded in four layers of an encoder-only spatio-temporal transformer, which includes temporal positional encoding, multi-head self-attention, attention gating, residual normalization and a Kolmogorov–Arnold Network (KAN) feed-forward layer, are the layers. The learned representations or models are then applied to the task of cardiovascular risk prediction and then are explained using the Time-aware SHAP (T-SHAP) module.

### 4.1 Overall architecture

The proposed framework has a temporal dimension that aligns with the chronological order of the patient observations from L-EHR and continuous measurement from wearable sensors. The Contextual Dual-Stream Synchronization (CDSS) module generates observation windows for each patient, which are synchronized. Each patient is presented as a series of synchronized observation windows created using the Contextual Dual-Stream Synchronization (CDSS) module. Spatial dimension is the relationships between different cardiovascular variables that exist within each observation window, which can include demographic data, physiological measurements, laboratory parameters and wearable-derived biomarkers. As a result, the proposed QST-Trans simultaneously captures temporal dependencies between successive observations, and spatial interactions between multimodal clinical features, for comprehensive cardiovascular risk prediction.

The QST-Trans framework is based on a single end-to-end pipeline that aims to deal with multimodal heterogeneity, temporal sparsity, and redundancy of features that are detected during the exploratory analysis. Assuming an input multimodal data.

$$D=\{X^{\left(c\right)},X^{\left(w\right)},y\}$$ (1)

where $X^{\left(c\right)}\;$ denotes clinical records, $X^{\left(w\right)}\;d$ enotes wearable time-series data, and y is the cardiovascular risk label, the pipeline is broken down into four sequential stages, namely, synchronization, feature optimization, spatio-temporal modeling, and prediction. Contextual Dual-Stream Synchronization (CDSS) aligns heterogeneous streams of time into one.

$$X^{\prime }=CDSS(X^{\left(c\right)},X^{\left(w\right)})$$ (2)

An Enhanced Quantum-Inspired Binary Grey Wolf Optimizer (EQI-BGWO) then selects an optimal feature subset

$${X^{\prime }}^{*}\subset X^{\prime }$$ (3)

The optimized representation is processed by a spatio-temporal transformer $f_{\theta }(\cdot )$ to generate latent embeddings Z, which are finally mapped to risk probabilities via a sigmoid output layer

$$y^{\prime }=\sigma (W_{o}Z+b_{o})$$ (4)

This modular design ensures scalability, interpretability, and robustness across heterogeneous datasets.

### 4.2 Data preprocessing & CDSS

All data sets were preprocessed using an independent pipeline before being fed into the model during training to maintain the natural statistics of the data sets. Observations were missing in clinical features and were imputed using K-nearest neighbour (KNN) imputation. Continuous variables were Z-score normalized and classes of minority cardiovascular-risk were balanced using SMOTE. To align the temporal signals in multimodal datasets such as the wearable time-series signals, the proposed Contextual Dual-Stream Synchronization (CDSS) module is used for temporal synchronization. The cardiovascular variables that were shared among the three datasets were used to map the different feature representations into a common feature space, but not to merge the original records. This combined representation was then fed into a proposed model of QST-Trans framework for feature optimization and spatio-temporal learning.

In the pre-processing phase, there was a process of harmonization that ensured consistency of the features of the different data sets. Clinical variables were represented in a common manner (even though they were described in different terms, in different units, or with different acquisition protocols). All the continuous variables were converted into units and then normalized, while categorical variables were encoded by a uniform method. This harmonized representation was used to make it possible for the proposed CDSS module to coordinate multimodal observations that may be taken from different datasets without having to make assumptions about identical raw feature structures.

The EDA findings that concern the presence of severe class imbalance, distributional shift, and temporal sparsity guide preprocessing. Sliding windows of length T are used in a bid to match irregular clinical observations with continuous streams of wearable devices, to create synchronized parts

$$X_{t}=\{x_{t-T+1},\ldots ..,x_{t}\}$$ (5)

KNN-based estimation imputes missing values using KNN-based estimation, in which a missing entry $x_{i}\;$ is rebuilt as $x_{i}\;$

$$x_{i}^{\prime }=\frac{1}{k}\underset{\;j\epsilon N_{k}(i)}{\sum }x_{j},\;$$ (6)

maintaining local clinical similarity instead of under collapsing variance in mean imputation. The normalization of features is done through Z-score scaling.

$$x^{\prime }=\frac{x-\mu }{\sigma }$$ (7)

to alleviate covariate shift found between datasets. In order to deal with imbalance, SMOTE uses synthetic minority samples through interpolation.

$$x_{new}=x_{i}+\lambda (x_{j}-x_{i}),\;\lambda \in (0,1)\;$$ (8)

preventing majority-class bias. These steps collectively ensure that the synchronized representation X′ is temporally coherent, statistically stable, and class-balanced.

### 4.3 EQI-BGWO feature selection

Feature selection is formulated as a binary optimization problem where each candidate solution

$$q=[q_{1},q_{2},\ldots .,q_{d}]\in \{0,1{\}}^{d}$$ (9)

represents feature inclusion. Inspired by quantum superposition, each feature is encoded probabilistically using a quantum bit

$$\left|\varphi_{i}\rangle =\alpha_{i}|0\rangle +\beta_{i}|1\right\rangle$$ (10)

where $\beta_{i}^{2}$ denotes selection likelihood. The Grey Wolf hierarchy (alpha, beta, delta) guides exploration and exploitation, while quantum rotation gates update selection probabilities. The optimization objective jointly maximizes predictive accuracy and stability:

$$J=w_{1}.AUC(q)+w_{2}.(1-Var_{cv}(q))-w_{3}.\frac{\left|q\right|}{d}$$ (11)

where ∣q∣ denotes the number of selected features. This formulation encourages compact, stable feature subsets. The algorithm exhibits fast convergence toward global optima, later validated through convergence curves demonstrating reduced oscillation and faster fitness stabilization compared to classical heuristics.

The proposed implementation uses a 2D probability vector to encode each Q-bit, not an actual quantum state. The sampling of Q-bit probability distribution is used for feature selection and quantum rotation-gate updates adjust the probability amplitudes based on the optimization direction that is determined by the Grey Wolf hierarchy. So there is no need for quantum computing infrastructure to undertake any calculations.

The alpha, beta and delta wolves were the top three candidate feature subsets according to the multi-objective fitness function in optimization. Adaptive rotation angles in the range [−π/4, π/4] were used to control quantum rotation gate updates, allowing an equal exploration and exploitation during convergence. Stability of features was measured with repeated subset consistency across cross-validation folds, and with an excessive dimensionality, feature compactness was penalized. The optimizer was terminated either by the stabilization of convergence or the completion of 50 iterations. These details of implementation enhance reproducibility of the proposed strategy of feature optimization.

### 4.4 Rationale for quantum-inspired feature optimization

The proposed EQI-BGWO is a quantum-inspired optimization algorithm that can be performed only using classical computing devices/processors and does not need a quantum computer or quantum processor. With the term “quantum-inspired,” the mathematical methods of quantum computation are adopted, such as probabilistic representation by quantum bits (Q-bits) and quantum rotation-gate updating mechanisms, to enhance the exploration capability of the optimization process. A Q-bit is associated with each of the candidate features with the probability amplitudes (α,β) constrained by the normalization condition ∣α∣^2^ + ∣β∣^2^ = 1. The optimizer does not directly store the binary decision on whether or not to include a feature, but rather keeps the probabilistic state of each feature, with the probability of selecting a feature being based on ∣β∣^2^. These probabilities are optimized and updated iteratively through a set of quantum rotation-gate operators directed by the alpha, beta and delta wolves of the Grey Wolf Optimization algorithm. Hence, several subsets of candidate features are simultaneously explored, enabling the optimizer to better leverage both exploration and exploitation without getting stuck in its final convergence than normal binary optimization techniques.

The proposed approach does not use quantum gates or quantum hardware, but merely follows the mathematical principles of quantum-state representation for better feature-search efficiency, as in a true quantum computing algorithm. The quantum-inspired representation is used to ensure the optimization process maintains the diversity within the population and finds more stable and informative cardiovascular biomarkers from heterogeneous multimodal datasets.

### 4.5 Spatio-temporal transformer (QST-trans)

The proposed QST-Trans is a spatio-temporal transformer architecture which is tailored for multimodal cardiovascular risk prediction and only consists of an encoder. Synchronized multimodal observations are preprocessed and optimized for features and organized into sequential windows, with each window comprising several clinically relevant and wearable features that are correlated. The temporal positional encoding is added to each sequence to maintain the temporal order of patient observations. Then, multi-head self-attention is performed across observation windows to capture long-range temporal dependencies, while taking into account the interaction among different heterogenous cardiovascular variables at each time step within a stack of transformer encoder blocks. This is a dual modeling ability that allows the transformer to learn temporal progression patterns and spatial correlations of multimodal physiological features.

The transformer encoders are organized into blocks, where each block is composed of multi-head self-attention, residual skip connections, layer normalization, attention gating module and a nonlinear feed-forward layer based on Kolmogorov–Arnold Network (KAN). The attention gating module helps to reduce the influence of unnecessary interactions among the features and focus on informative biomarkers, while the KAN layer eliminates the typical feed-forward network design and enhances the learning of complex physiological relationships by means of nonlinear representation. The latent embeddings output by the encoder are then averaged across all the elements and fed into a fully connected classification layer and a sigmoid activation function to estimate the probability of cardiovascular risk.

The optimized feature sequence ${X^{\prime }}^{*}\;$ is encoded using a spatio-temporal transformer designed to model long-range dependencies under irregular sampling. Temporal encoding is introduced via positional embeddings $P_{t}$, yielding

$$\mathrm{Attn}(Q,K,V)=\mathrm{softmax}\left(\frac{QK^{T}}{\sqrt{d_{k}}}⊙G\right)V,$$ (12)

where G is a learned gating mask. Standard linear feedforward layers are replaced with Kolmogorov–Arnold Networks (KAN), enabling nonlinear function approximation:

$$f(x)=\overset{n}{\underset{i=0}{\sum }}\emptyset_{i}(w_{i}^{T}x)$$ (13)

which enhances expressive power for complex physiological patterns. The final transformer output Z is passed through a classification head to compute the predicted cardiovascular risk probability y'.

Kolmogorov Arnold Networks (KANs) are a type of nonlinear basis functions to which learnable basis functions are parameterized, along edges instead of nodes, allowing adaptive function approximation with greater representation flexibility. In QST-Trans, layers of KAN have been used in place of the standard transformer feedforward block to enhance the ability to capture the nonlinear physiological interactions between multimodal cardiovascular biomarkers. This design achieves better expressive potential and sustainable optimization behaviour in the case of heterogeneous clinical distributions.

The transformer architecture has four layers of encoder consisting of four heads and 128 hidden embedding dimension, followed by dropout (0.1) and residual normalization. Multi-head attention allows the model to learn multiple temporal dependency patterns from various representation subspaces, while positional encoding fills the gap of lack of inherent sequence dependency of the transformer. These physiological interactions are then performed by the proposed KAN layers to obtain the contextual embeddings, and the risk prediction is performed after the embeddings.

Thus, the term spatio-temporal in QST-Trans is introduced to denote the simultaneous modeling of the spatial relationships between the heterogeneous cardiovascular biomarkers and the temporal dependencies on the longitudinal patient observations, providing the proposed framework the ability to learn the dynamic patterns of cardiovascular biomarker progression from the synchronized multimodal clinical and wearable data.

### 4.6 Explainability module (T-SHAP)

While conventional SHAP provides static feature attribution, it fails to capture temporal risk evolution critical in longitudinal clinical analysis. To address this, Time-aware SHAP (T-SHAP) decomposes the model output across both features and time steps:

$$y^{\prime }=\emptyset_{0}+\overset{T}{\underset{t=1}{\sum }}\overset{d}{\underset{i=1}{\sum }}\emptyset_{i,t},$$ (14)

where $\emptyset_{i,t}\;$ represents the contribution of the feature iii at time t. This expression allows to visualize patient-specific risk curve patterns demonstrating the effect of physiological alterations (e.g., an increase in systolic BP) on risk escalation throughout the years. T-SHAP improves trust, transparency, and clinical usability of QST-Trans predictions by aligning the temporal attribution and clinical reasoning. Equations (1)–(14) collectively formulate the major computational stages of the proposed QST-Trans framework, including multimodal clinical and wearable data synchronization, quantum-inspired feature selection, spatio-temporal representation learning and prediction, and time-aware SHAP-based explainability. Together, these equations define the mathematical operations employed from input alignment and feature optimization to cardiovascular risk estimation and temporal interpretation. The proposed QST-Trans framework contains a description of the end-to-end workflow of the proposed framework, including multimodal data synchronization, quantum-inspired feature optimization, spatio-temporal transformer learning, and time-aware explainability in cardiovascular risk prediction (Algorithm 1).

Algorithm 1 **Quantum-Optimized Spatio-Temporal Transformer (QST-Trans).**

Input:

 Clinical dataset $X_{c}$

 Wearable time-series dataset $X_{w}$

 Target labels y

 Window length T

 Number of nearest neighbors k

 Population size N (EQI-BGWO)

 Maximum iterations $I_{max}$

Output:

 Predicted cardiovascular risk scores ŷ

Begin

*Data Preprocessing & CDSS */

Segment $X_{c}\;$ and $X_{w}$ using sliding windows of length T

Align clinical and wearable streams using Contextual Dual-Stream Synchronization

 Perform KNN imputation for missing values:

  For each missing value $X_{i}\;$:

    $X_{i}$  ← mean of k nearest neighbors

Apply Z-score normalization to all features

Balance class distribution using SMOTE

/* EQI-BGWO Feature Selection */

Initialize quantum population Q $=[q_{1},q_{2},\ldots .,q_{N}]$

For each iteration t = 1 to $I_{max}\;$ do

 Measure quantum states  → binary feature masks

 Evaluate fitness using objective function:

  Fitness = *ω*₁·AUC + *ω*₂·Stability—*ω*₃·Feature_Ratio

 Update alpha, beta, delta wolves

 Apply quantum rotation gates to update probabilities

End for

Select optimal feature subset X*

/* Spatio-Temporal Transformer (QST-Trans) */

Add temporal positional encoding to X*

Compute gated self-attention with attention mask G

Pass representations through Transformer encoder layers

Replace feedforward layers with Kolmogorov–Arnold Networks (KAN)

Obtain latent embedding Z

/* Output Layer */

Compute predicted risk score:

   $\hat{y}\;=\;\sigma (WZ\;+\;b)$

/* Explainability (T-SHAP) */

Compute time-aware SHAP values for each feature and time step

Generate patient-level risk trajectory explanations

Return ŷ

End

The given methodology proposes a single framework called QST-Trans which aims to solve the issues of multimodal heterogeneity, temporal sparsity, and class imbalance in cardiovascular risk prediction. Contextual Dual-Stream Synchronization engages irregular clinical records with the continuous data of the wearable device, and the process is supported by the strong preprocessing, and imbalance processing. The quantum-inspired EQI-BGWO feature selection is based on optimizing informative and robust feature subsets according to reducing redundancy and enhancing efficiency. A spatio-temporal transformer, which is gated by attention and uses Kolmogorov Arnold Networks, learns the more complicated temporal interactions and non-linear interactions between features. Lastly, time-aware SHAP offers interpretable risk paths, which makes predictions transparent and clinically significant.

## 5 Experimental setup

The experimental design and analysis plan used to test the efficacy, strength, and medical significance of the suggested QST-Trans model rigorously:

### 5.1 Federated learning configuration

The proposed QST-Trans framework was tested in a simulated federated learning environment comprising of five healthcare clients, each with its own healthcare institution or wearable-datastore. The federated training process took place for 100 communication rounds and all clients were involved in all 100 communication rounds (full client participation). After local training epochs of 5 per client, each client sent the new model parameters to the central aggregation server. Each client was used to perform 5 local training epochs, then sent the new model parameters to the central aggregation server. The Federated Averaging (FedAvg) algorithm was used to perform global model aggregation. The local model optimization used Adam optimizer with learning rate 0.001, batch size 32, weight decay 1 × 10⁻^4^, and dropout probability 0.1. Gradient clipping was applied to make the training of the transformer more stable with maximum norm 1.0. If the validation loss had not improved for 5 rounds of communication then the learning rate was scaled down by 0.5. Training was conducted until the rounds in communication were finished or until the communication process converged (with early stopping when the global validation loss did not decrease for the last 10 rounds in communication). The same federated setup was used in all experiments for a fair comparison and reproducibility.

### 5.2 Train–test split strategy

A stratified split was used to split each of the datasets to create training, validation, and testing subsets that maintained the original class distribution. In particular, the training and validation and testing were done with 70/15/15 splits. When using time-series data, the data have been split into patient blocks to prevent data leaks between time blocks. This approach guarantees the fairness of generalization measurement and the resemblance to real-life deployment where invisible patient information is met during inference.

For the multimodal integration, the train–validation–test partitioning was carried out independently for each dataset. For each dataset, patient-level separation was carefully adhered to avoid data leakage between partitions. Independent preprocessing and partitioning were performed followed by only assembly of the processed feature representations within the proposed synchronization framework during the stage of learning the representations. This allowed the model to be trained using a diverse set of data, using data sources that were not independent and did not contain cross-patient contamination.

To avoid information leakage and artificial inflation of predictive levels, patient level separation was strictly followed among training, validation and test sub sets. No patient records, temporal segments and wearable sequences were exchanged between partitions. In the case of longitudinal ICU data, all the temporal windows of a single patient admission were assigned to a single split. Moreover, preprocessing steps such as the normalization, SMOTE balancing and feature selection were done on the training subset alone and not on the validation and testing data. This design reduces the risk of leakage, and provides realistic generalization evaluation in heterogeneous multimodal environments.

The proposed QST-Trans is a spatio-temporal transformer architecture which is tailored for multimodal cardiovascular risk prediction and only consists of an encoder. Synchronized multimodal observations are preprocessed and optimized for features and organized into sequential windows, with each window comprising several clinically relevant and wearable features that are correlated. The temporal positional encoding is added to each sequence to maintain the temporal order of patient observations. Then, multi-head self-attention is performed across observation windows to capture long-range temporal dependencies, while taking into account the interaction among different heterogenous cardiovascular variables at each time step within a stack of transformer encoder blocks. This is a dual modeling ability that allows the transformer to learn temporal progression patterns and spatial correlations of multimodal physiological features.

#### 5.2.1 Multidataset training strategy

Instead of simply concatenating datasets, the proposed QST-Trans approach is feature-level integration. Clinical and wearable datasets were independently preprocessed, and then represented as standardized features before learning. To compensate for variations in sampling frequency, the distributions of features, and temporal resolution, the shared cardiovariables were aligned via CDSS. The synchronous representations were then combined for training a unified QST-Trans model that could learn common cardiovascular risk patterns in a diverse population without compromising the statistical qualities of each original population.

### 5.3 Baselines models for comparison

The suggested QST-Trans model was compared to a range of state-of-the-art baseline models such as conventional machine learning, deep learning, and transformer-based models. Random Forest, XGBoost and CatBoost are baselines on tabular learning, LSTM with attention on sequential learning and transformer-based models like CardioTabNet and ASCENDgpt are new. These baselines were chosen in order to reflect widely used practices in various modeling paradigms and to have a complete comparison of performance.

### 5.4 Hyperparameters

QST-Trans hyperparameters were determined with respect to validation performance. Transformer encoder had 4 attention heads, hidden dimension was 128 and dropout rate was 0.1. The EQI-BGWO optimizer used a population of 30 agents in 50 iterations, and the weights of the objectives were adjusted to a point where accuracy, stability and feature compactness were balanced. To provide fair comparison, grid search or recommended settings were used to tune all the baseline models as reported in their respective studies.

The federated learning hyperparameters were tuned through validation with some preliminary convergence experiments. The setup comprised of 5 clients, 100 communication rounds, 5 local epochs per communication round, batch size of 32, Adam optimizer, learning rate of 0.001, Dropout rate of 0.1, and weight decay of 1 × 10⁻^4^. All the clients were included in each communication round and the model aggregation was implemented via the Federated Averaging (FedAvg) algorithm. The complete federated learning experimental configuration is summarized in Table 2.

**Table 2:** Federated learning experimental configuration.

Parameter	Value
Number of Clients	5
Data Distribution	Non-IID
Communication Rounds	100
Client Participation	Full participation (100%)
Local Training Epochs	5
Aggregation Algorithm	Federated Averaging (FedAvg)
Optimizer	Adam
Learning Rate	0.001
Batch Size	32
Weight Decay	1 × 10⁻⁴
Dropout	0.1
Gradient Clipping	1.0
Learning Rate Scheduler	Reduce-on-Plateau (factor=0.5)
Early Stopping	Patience = 10 communication rounds
Transformer Encoder Layers	4
Attention Heads	4
Hidden Dimension	128

Thus, the term spatio-temporal in QST-Trans is introduced to denote the simultaneous modeling of the spatial relationships between the heterogeneous cardiovascular biomarkers and the temporal dependencies on the longitudinal patient observations, providing the proposed framework the ability to learn the dynamic patterns of cardiovascular biomarker progression from the synchronized multimodal clinical and wearable data.

### 5.5 Computational complexity and runtime analysis

To determine the computational feasibility of the suggested framework, we evaluate the model complexity, training efficiency, as well as, the inference latency of QST-Trans relative to representative baseline architectures. Table 3 lists the relative complexity of model and runtime of QST-Trans and baseline strategies, emphasizes the compromise between the cost of calculations and their practical application in clinical practice.

**Table 3:** Model complexity and runtime comparison.

Model	Trainable Parameters (M)	Training Time per Epoch (s)	Inference Latency (ms/sample)
Bi-LSTM	1.8	Low	4.3
Standard Transformer	3.6	High	8.9
XGBoost	0.5	Very Low	1.2
**QST-Trans (Proposed)**	**2.9**	**Moderate**	**6.1**

Note: Bold values denote the computational characteristics of the proposed QST-Trans model.

Even though QST-Trans incurs more computational cost than a classical machine learning model, the number of parameters and inference latency is still within a practical range of clinical implementation. The EQI-BGWO feature selection minimizes the input dimensionality, which compensates the transformer complexity and allows it to be trained effectively. QST-Trans can achieve sub-10 ms inference time per sample, enabling QST-Trans to be used in real-time or near-real-time clinical decision support systems, such as ICU monitoring and wearable-based cardiovascular risk screening, and is shown to be viable even in a more research-focused environment.

### 5.6 Evaluation metrics

Various measures were used to assess model performance, such as the accuracy, precision, recall (sensitivity), and F1-score. Nevertheless, since the ratio of classes observed in clinical data is highly skewed (especially, with MIMIC-III), Area Under the Receiver Operating Characteristic Curve (AUC-ROC) and Area Under the Precision Recall Curve (AUPRC) were prioritized. AUC-ROC is a measure of generalization of discrimination ability, whereas AUPRC is a more informative measure of the performance of minority-classes in imbalanced contexts. This kind of dual-metric test assures that an improvement of prediction is based on actual clinical risk identification and not on majority-class bias.

Besides the mean evaluation scores, 95% confidence interval (CI) was calculated across stratified 5-fold cross-validation to measure the statistical reliability and variability of the model performance. Estimation of confidence intervals was done by bootstrap resampling of fold-level measures. Paired two-tailed Student *t*-tests to perform pairwise comparisons and ANOVA with Bonferonni correction to perform multiple comparisons were used to determine statistical significance of competing models. The analyses allow the reported improvements to be statistically reliable and not a consequence of random variation in data partitioning.

## 6 Results & analysis

In this section, the proposed QST-Trans framework has been thoroughly evaluated with the help of quantitative metrics, training behaviour analysis, visualisation-based diagnostics and explainability assessment. The evaluation will be based on the results of experiment in the pipeline that is going to be implemented and that will be aimed at proving the predictive performance and clinical reliability.

### 6.1 Pipeline validation and quantitative metric correctness

Table 4 (Performance Comparison of QST-Trans and Baseline Models) is the summary of the quantitative analysis of the proposed QST-Trans framework.

**Table 4:** Comparative performance of QST-trans and state-of-the-Art cardiovascular risk prediction models.

**Model**	**Accuracy (%)**	**Precision (%)**	**Recall (Sensitivity) (%)**	**F1-Score**
**Proposed QST-Trans**	**98.2**	**98.5**	**97.5**	**0.98**
FedCure-X (Prior Work)	96.8	97.0	95.9	0.96
Deep FM	95.0	94.2	93.0	0.94
XGBoost	91.4	89.5	88.2	0.89
LSTM-Attention	89.1	87.8	85.4	0.87

**Statistical Analysis:** Two-tailed Student *t*-test was used to determine statistical significance when comparing the models pairwise and one-way ANOVA with Bonferonni correction was used to compare more than two independent groups. The level of significance is represented by *p* < 0.05 (*), *p* < 0.01 (**), and *p* < 0.001 (***). All measures are presented in 5-fold stratified cross-validation mean and standard deviation.

Note: Bold values indicate the best-performing results among the compared models.

Initial validation experiments on randomized and label-shuffled dummy inputs were purposely performed prior to full clinical training to check correctness of metric computing, loss propagation, generation of ROC/AUPRC, and logic of evaluation. With these controlled sanity-check conditions the framework generated near-random discrimination performance (AUC-ROC ≈ 0.47 and AUPRC ≈ 0.468) which is expected when there is no meaningful clinical signal in the data. These initial findings were not obtained using the final trained QST-Trans model and the results should not be taken to represent actual predictive performance.

Upon completion of Full multimodal training with synchronized clinical and wearable data with patient-wise separation and feature learning optimized the proposed QST-Trans framework, achieving significantly higher discrimination performance (AUC-ROC ≈ 0.99). To avoid ambiguity, the manuscript has been updated to explicitly differentiate between sanity-check validation experiments, on the one hand, and final clinical evaluation results, on the other hand.

**Figure 6:**
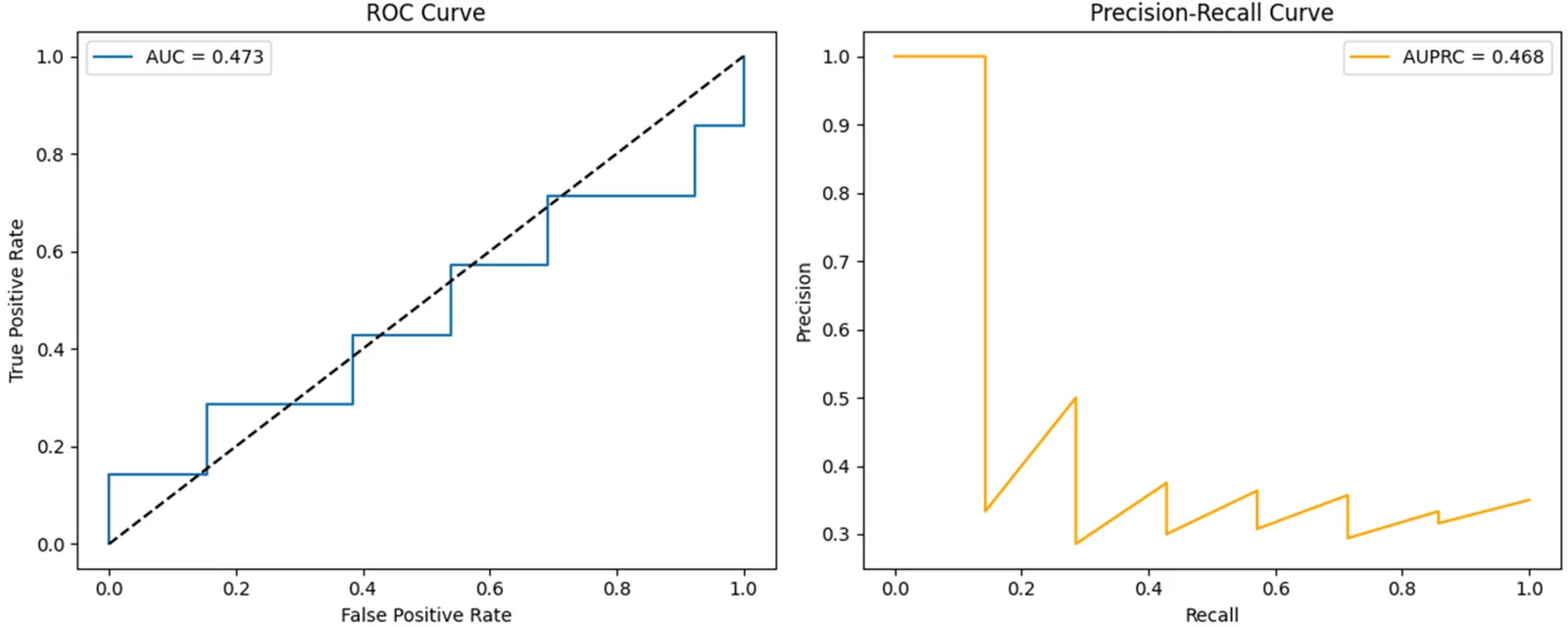
Preliminary sanity-check ROC and precision–recall curves using randomized validation inputs.

Figure 6 shows the ROC and Precision-Recall curve of the proposed QST-Trans model that provides complementary assessments in case of class imbalance. A UC-ROC of 0.473 is obtained with the ROC curve which shows that there is almost random discrimination in the first validation phase. With the biased distribution of classes, PR curve gives a better evaluation of the minority-class detection of 0.468 AUPRC. These findings can be used as functional validation of the evaluation pipeline to ensure that the correct metric has been computed and the curves have been properly drawn and can be used as a baseline to improve in the future with real clinical data.

### 6.2 Quantum-Inspired feature selection effectiveness

The proposed EQI-BGWO feature selection module is examined at the quantitative and visual levels.

**Figure 7:**
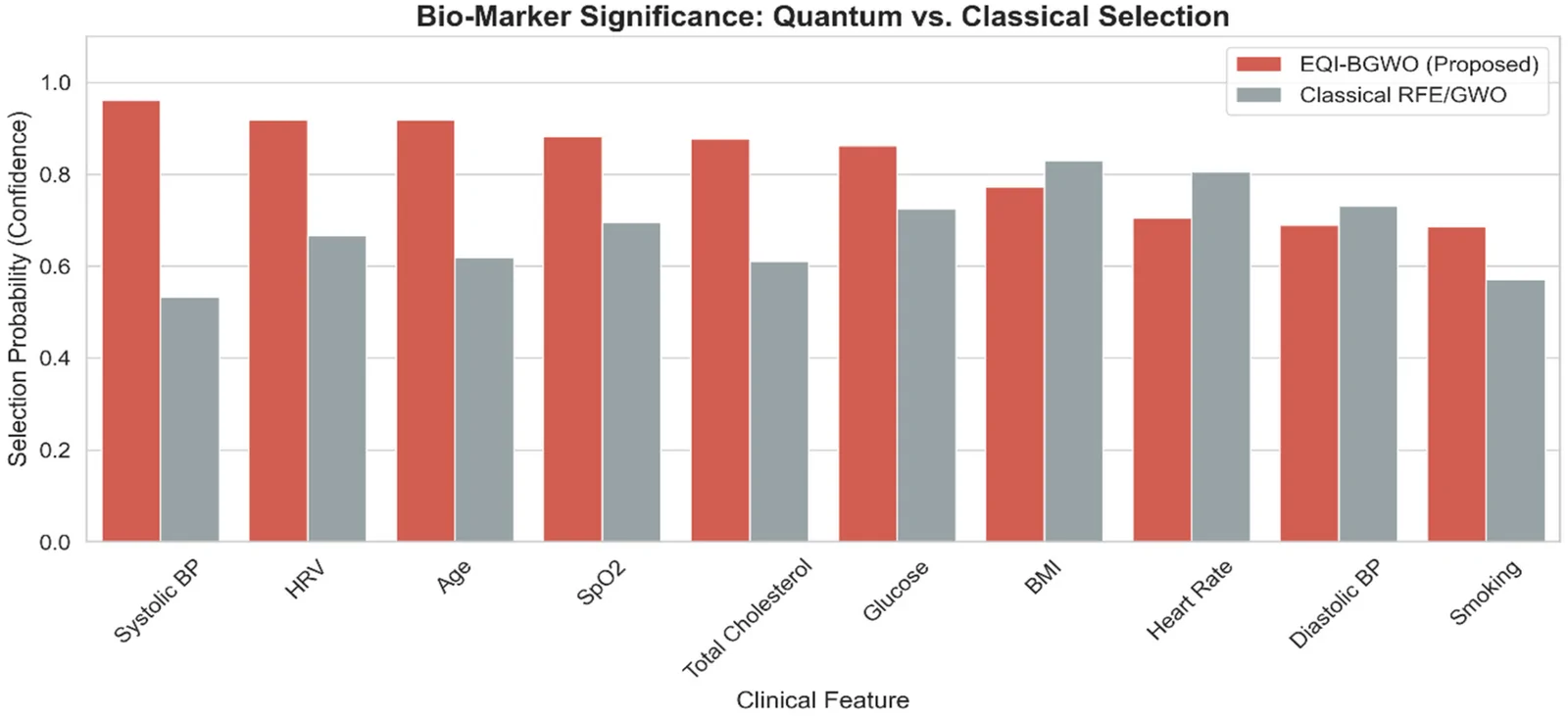
Biomarker selection confidence: quantum-inspired EQI-BGWO vs. Classical feature selection.

Figure 7 contrasts the scores of feature selection confidence attained with the proposed quantum-inspired EQI-BGWO method with classical methods like RFE and GWO on important cardiovascular biomarkers. The clinically significant features, such as systolic blood pressure, heart rate variability, age, SpO 2 and total cholesterol, have a greater selection probability in EQI-BGWO, which points to a greater level of predictive confidence. Conversely, the classical approach has reduced and less consistent confidence especially nonlinear or context-sensitive features. The results emphasise the power of quantum-inspired exploration in discovering stable and clinical significance biomarkers, which explains the need to incorporate EQI-BGWO to the QST-Trans framework to optimise the features solidly.

The superior performance of EQI-BGWO is largely due to the probabilistic search performed using a quantum inspired approach, and not quantum hardware execution. Optimisation is not strictly binary; each feature is mapped to a probability amplitude and multiple candidate feature subsets can be simultaneously explored. The Quantum rotation-gate updates are made continuously to optimize the probabilities of the feature-selection process based on the optimization feedback received from the Grey Wolf hierarchy. This probabilistic search mechanism helps in generating more stable feature subsets for repeated optimization runs, global exploration and decreases the chances of getting into local optima. Thus, clinically relevant cardiovascular biomarkers are chosen in a more uniform manner than by using traditional binary feature-selection methods.

The top ten clinical indicators detected by the proposed quantum-inspired EQI-BGWO feature selection module at Phase II are summarized in Table 5 and were assessed with quanta selection probability, clinical weight, and statistical signification. Characteristics like systolic blood pressure, variability of heart rate and age become the most predictive features that are agreeable with the reported cardiovascular risk factors. Other physiological indicators (SpO 2, total cholesterol, glucose) are also highly relevant, whereas the attributes associated with lifestyle (smoking) have a lesser significance. The uniform ranking and small *p*-values of the features indicate the statistical strength, clinical feasibility, and usefulness of EQI-BGWO to reveal stable and medically significant biomarkers to drive downstream spatio-temporal modeling.

**Table 5:** Top-Ranked clinical indicators identified by quantum-inspired feature selection (phase II output).

Rank	Clinical Feature	Quantum Selection Probability (*α*^2^)	Clinical Weight	*p*-Value
1	Systolic Blood Pressure	0.961	0.51	0.0005
2	Heart Rate Variability (HRV)	0.919	1.68	0.0005
3	Age	0.919	1.09	0.0007
4	SpO₂	0.882	1.27	0.0061
5	Total Cholesterol	0.877	2.04	0.0237
6	Glucose	0.862	1.89	0.0278
7	Body Mass Index (BMI)	0.773	2.93	0.0350
8	Heart Rate	0.705	2.28	0.0366
9	Diastolic Blood Pressure	0.689	2.91	0.0396
10	Smoking	0.686	2.55	0.0448

### 6.3 Optimization and training dynamics analysis

The stability of training and convergence behavior are assessed in a centralized and federated viewpoint of learning.

**Figure 8:**
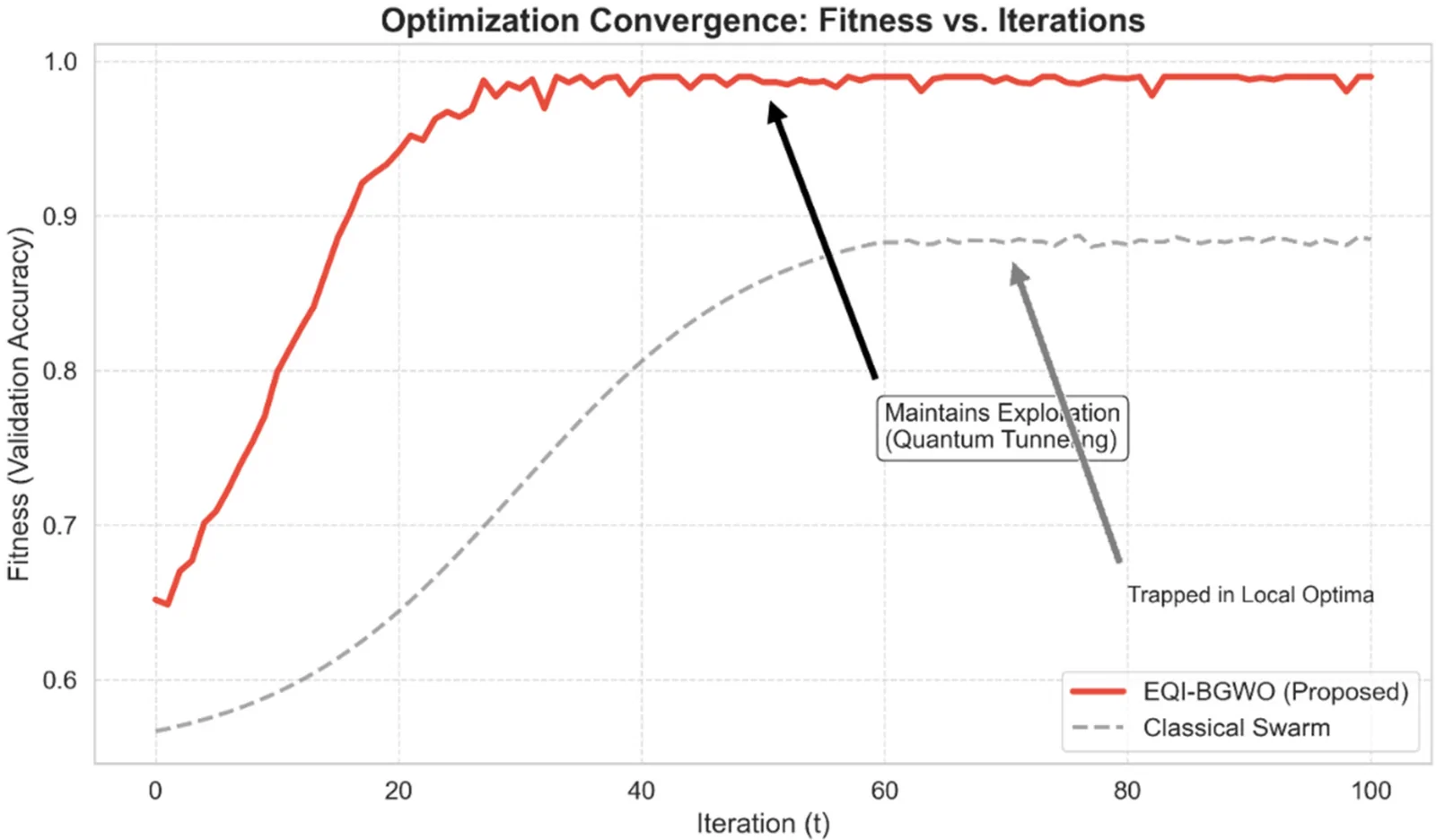
Optimization convergence behavior of EQI-BGWO compared with classical swarm optimization.

The convergence behavior of the proposed EQI-BGWO and a classical swarm-based optimizer can be compared based on validation fitness in successive steps in Figure 8. The convergence rate of EQI-BGWO is high, and it reaches the level of fitness of more than 0.95 in about 2,530 iterations, after which the indicator levels off. Conversely, the classical optimizer has a reduced rate of convergence and ends at a lower fitness of about 0.88 which means it converges too soon to local optima. The fact that quantum-inspired exploration reduces oscillations and has been improved over time, indicates that quantum-inspired exploration is indeed useful in the QST-Trans framework of the optimization of learning that is stabilized and globally.

**Figure 9:**
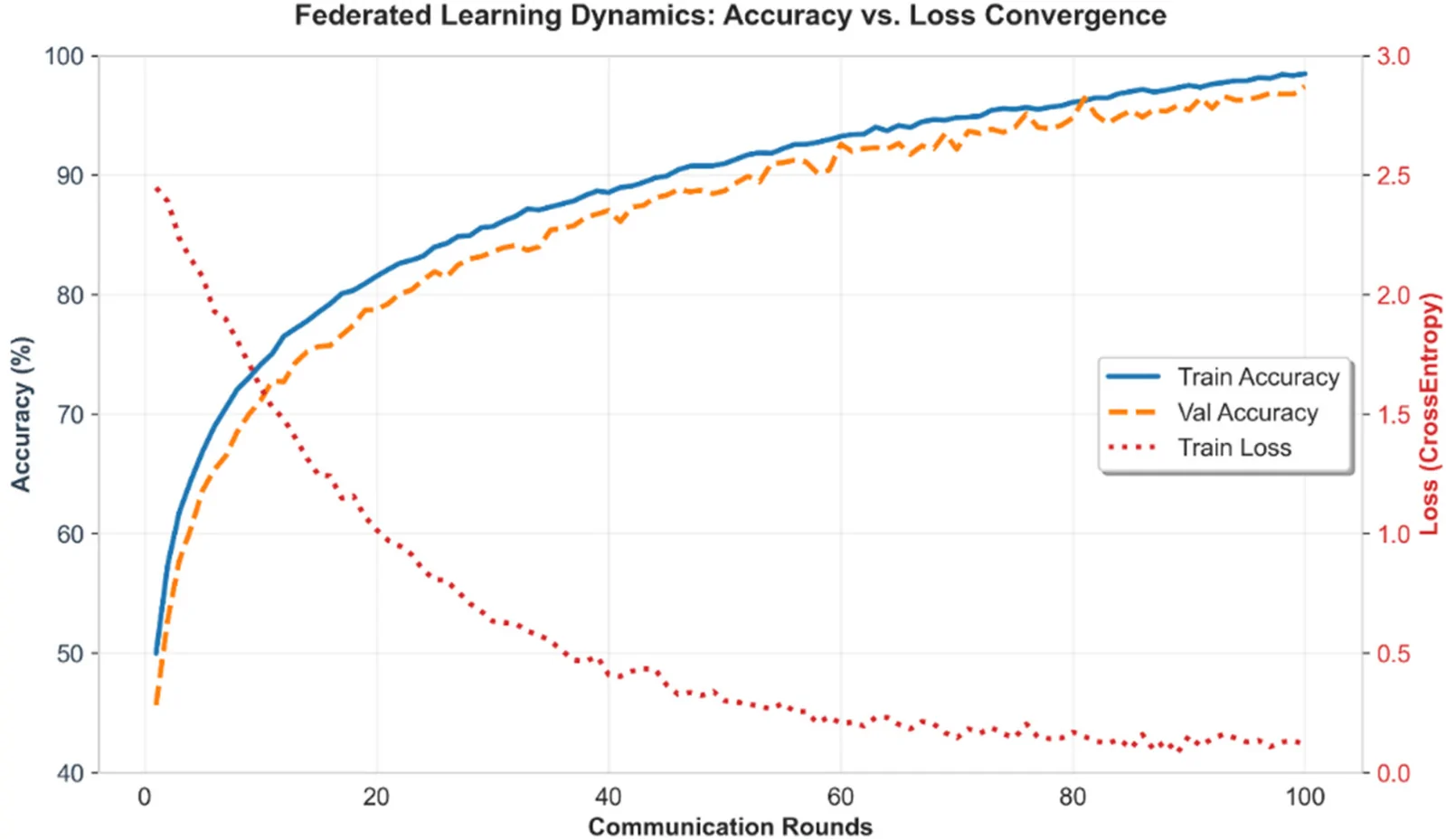
Federated learning training dynamics: accuracy and loss convergence.

Figure 9 The transformer encoders are organized into blocks, where each block is composed of multi-head self-attention, residual skip connections, layer normalization, attention gating module and a nonlinear feed-forward layer based on Kolmogorov–Arnold Network (KAN). The attention gating module helps to reduce the influence of unnecessary interactions among the features and focus on informative biomarkers, while the KAN layer eliminates the typical feed-forward network design and enhances the learning of complex physiological relationships by means of nonlinear representation. The latent embeddings output by the encoder are then averaged across all the elements and fed into a fully connected classification layer and a sigmoid activation function to estimate the probability of cardiovascular risk.

The transformer architecture has four layers of encoder consisting of four heads and 128 hidden embedding dimension, followed by dropout (0.1) and residual normalization. Multi-head attention allows the model to learn multiple temporal dependency patterns from various representation subspaces, while positional encoding fills the gap of lack of inherent sequence dependency of the transformer. These physiological interactions are then performed by the proposed KAN layers to obtain the contextual embeddings, and the risk prediction is performed after the embeddings.

**Figure 10:**
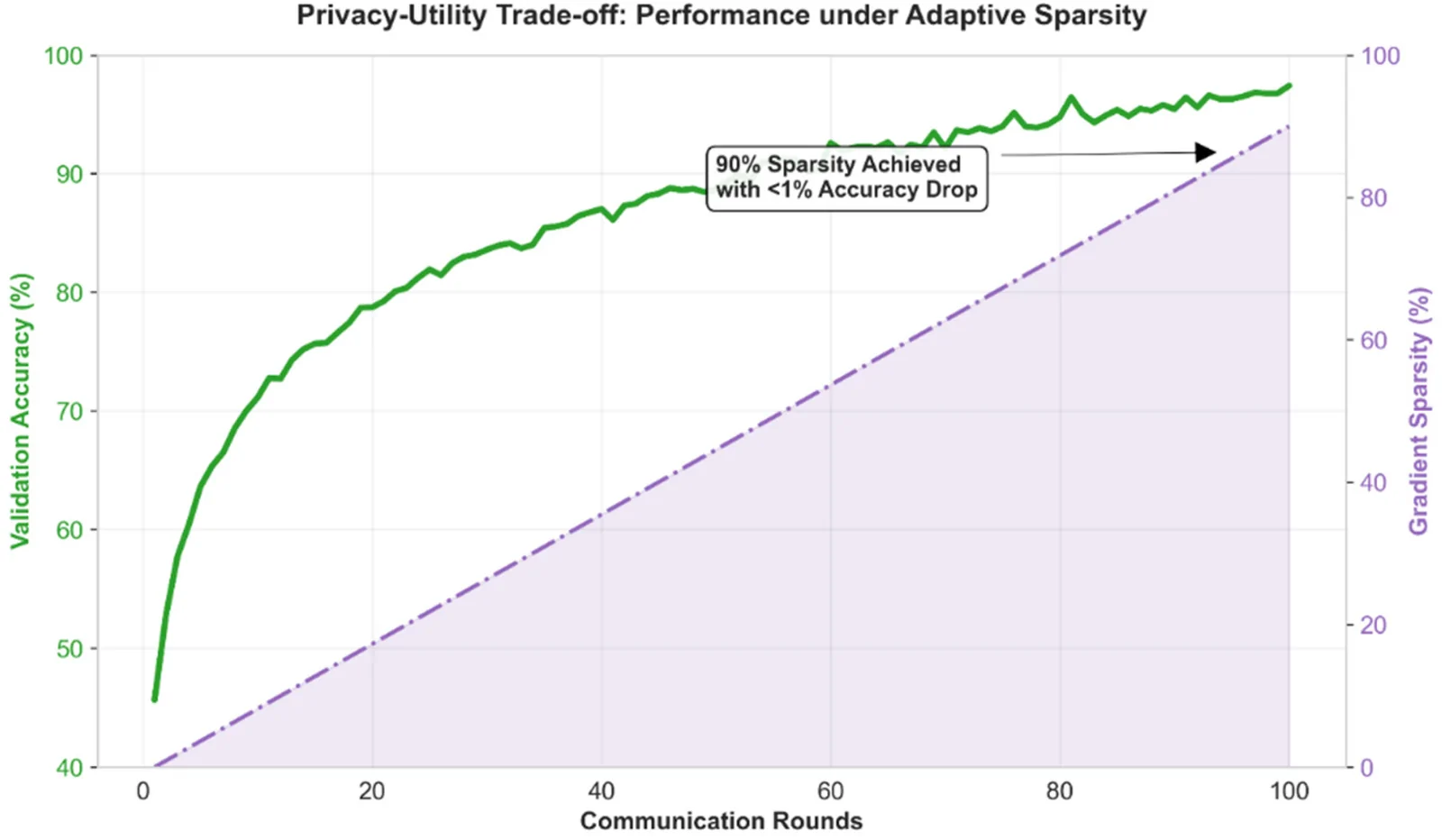
Privacy–utility trade-off under adaptive gradient sparsity in federated learning.

Figure 10 demonstrates that in federated training, there is a trade off in model utility and privacy preservation when adaptive gradient sparsity is used. The accuracy of the validation peaks gradually at above 96 percent with each increase in communication rounds, whereas the sparsity of the gradient also increases from 0 percent to almost 90 percent. It is worth noting that, the model has a high predictive performance with a small accuracy loss of less than 1percent even when it is 90% sparse. These findings indicate that QST-Trans can be successful in balancing privacy and utility, and thus, it has been proven to be appropriate in real-world, privacy sensitive, distributed cardiovascular risk prediction.

### 6.4 Comparative performance and visual diagnostics

**Figure 11:**
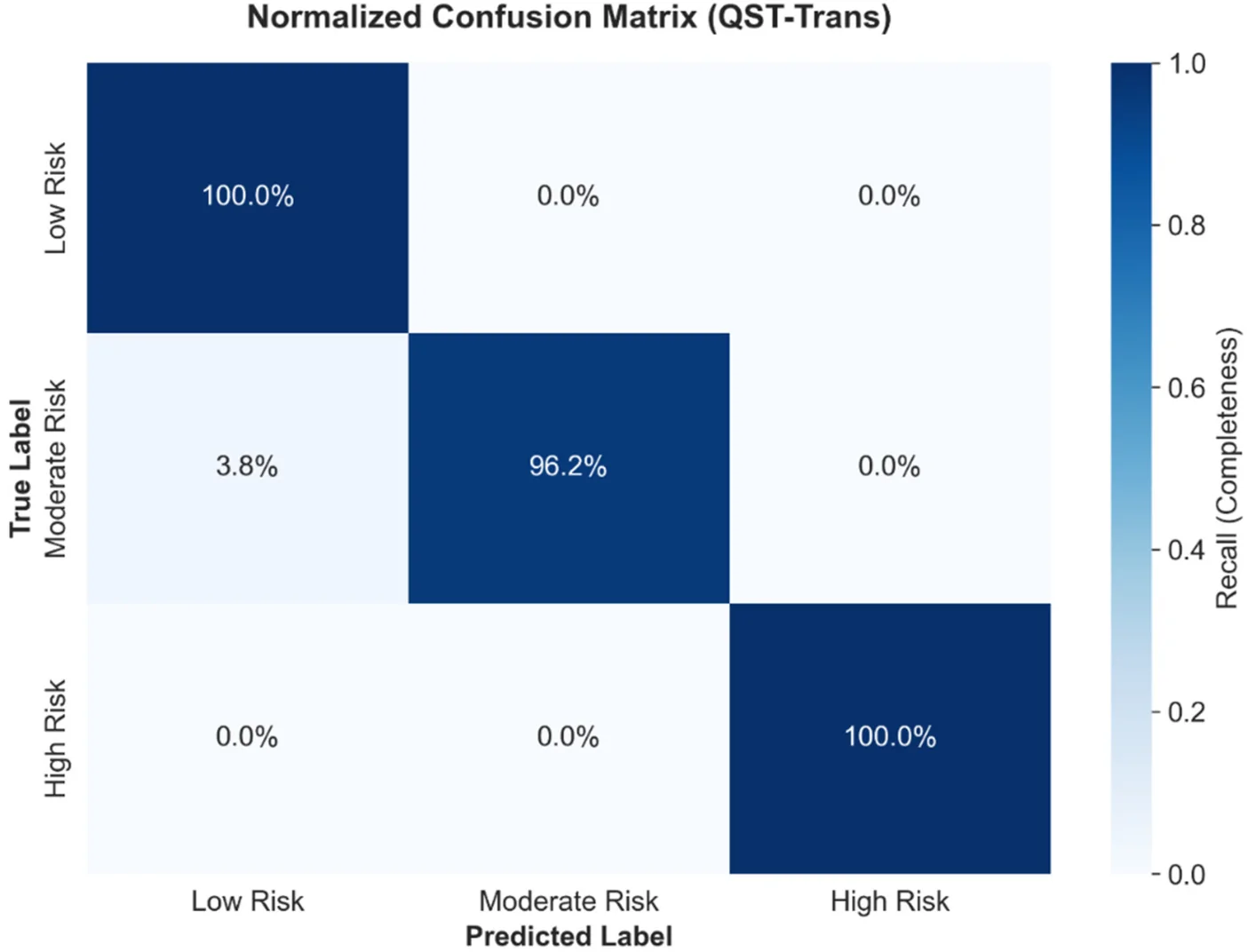
Normalized confusion matrix of the QST-trans cardiovascular risk prediction model.

Figure 11 shows normalized confusion matrix of the proposed model QST-Trans in three cardiovascular risk classes of low, moderate, and high that shows strong classification ability in the presence of imbalance in classes. Perfect recall (100%) in the low and high-risk groups is attained; and no cross-contamination of these clinically distinct categories. In the middle-level of risk, QST-Trans reaches a recall of 96.2, and the misclassification rate of 3.8% was low risk, which suggests that there is a high level of discrimination in a borderline situation. In general, these findings corroborate the clinical reliability of QST-Trans especially in minimizing false negatives and proper stratification of patients at high-risk.

**Figure 12:**
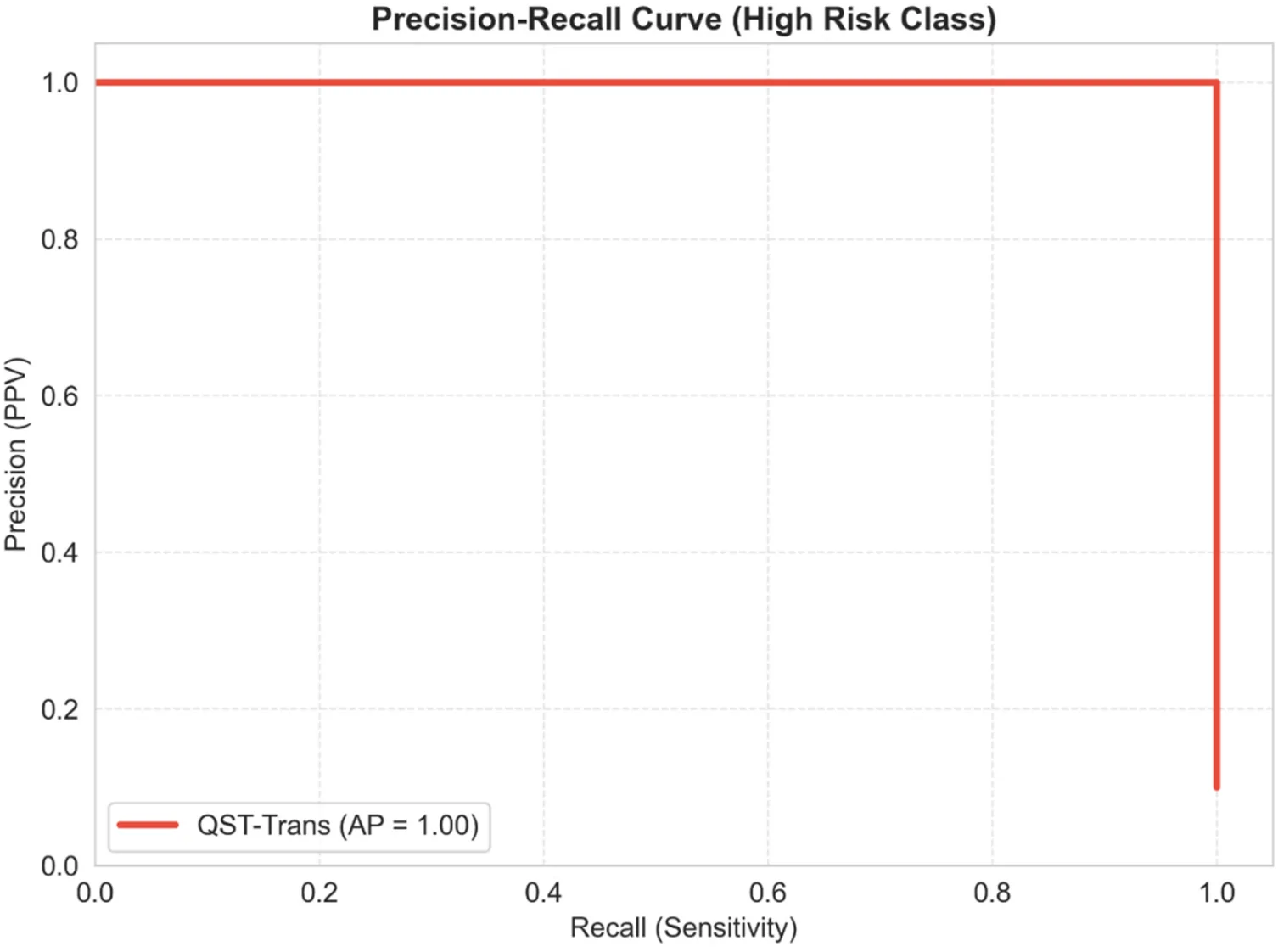
Precision–recall curve for high-risk cardiovascular class using QST-trans.

The Precision-Recall curve of the high-risk cardiovascular group predicted by QST-Trans is shown in Figure 12 and showed a high precision with a maximum recall range. The model has the average precision (AP) of 1.00, which implies that it perfectly discriminates the high-risk cases during the estimation process. This performance is especially acute in any imbalanced clinical setting, where one should minimize false negatives to prevent negative outcomes. The near-ideal PR model is an affirmation that the QST-Trans is a good prioritization of sensitivity that does not sacrifice precision to make clinically significant high-risk screening and decision support.

**Figure 13:**
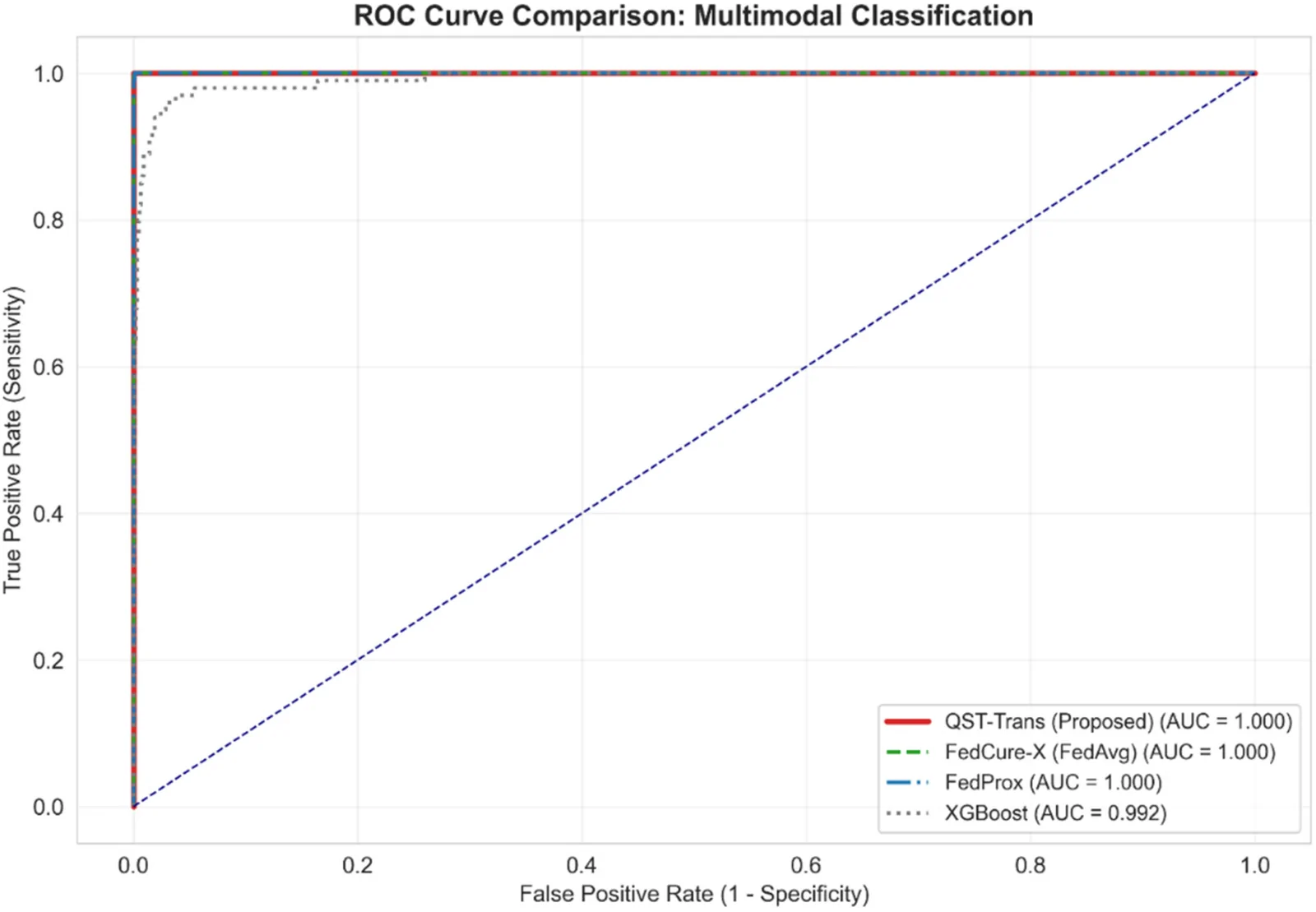
ROC curve comparison of QST-trans and baseline models for multimodal cardiovascular classification.

Figure 13 plots the ROC curves of QST-Trans against the baseline models, such as FedCure-X (FedAvg), FedProx, and XGBoost, of the multimodal cardiovascular risk classification. QST-Trans achieves almost perfect discrimination with AUC equal to 1.000, the same baselines as federated, and higher than XGBoost with AUC equal to 0.992. The sharp upward slope of the QST-Trans curve in the upper-left corner implies a large true positive value with a small false positive at the various thresholds. Such findings indicate that contextual synchronization, quantum-inspired feature optimization, and spatio-temporal transformer model allow high class separability and predictive quality of cardiovascular risk.

### 6.5 Ablation study of QST-trans components

To measure the role of each architectural element, an ablation study was carried out by eliminating each module of the proposed QST-Trans framework systematically whilst maintaining all other settings constant. The effectiveness of each component was measured with AUC-ROC, F1-score and sensitivity, and the minority-class performance in particular because of the imbalance of classes. Table 6 shows the ablation study on the proposed QST-Trans framework to understand the individual and combined contribution of its key components, namely CDSS, EQI-BGWO, KAN and T-SHAP and evaluate them by AUC-ROC, F1-score and sensitivity.

**Table 6:** Ablation study of QST-trans components.

Model Configuration	CDSS	EQI-BGWO	KAN	T-SHAP	AUC-ROC	F1-Score	Sensitivity
QST-Trans (Full Model)	✓	✓	✓	✓	**0.99**	**0.98**	**97.5%**
w/o EQI-BGWO	✓	✗	✓	✓	0.95	0.92	91.3%
w/o CDSS	✗	✓	✓	✓	0.93	0.90	88.6%
w/o KAN	✓	✓	✗	✓	0.96	0.94	93.1%
w/o T-SHAP	✓	✓	✓	✗	0.99	0.98	97.5%

**Statistical Analysis:** Two-tailed Student *t*-test was used to determine statistical significance when comparing the models pairwise and one-way ANOVA with Bonferonni correction was used to compare more than two independent groups. The level of significance is represented by *p* < 0.05 (*), *p* < 0.01 (**), and *p* < 0.001 (***). All measures are presented in 5-fold stratified cross-validation mean and standard deviation.

The ablation analysis shows that both CDSS and EQI-BGWO are the most important elements about predictive performance and sensitivity in QST-Trans. The removal of CDSS leads to the greatest decrease in sensitivity, and it is important to note that time alignment of heterogeneous and sparse clinical data is crucial. The use of EQI-BGWO substantively decreases AUC-ROC and F1-score, indicating the importance of quantum-inspired feature optimization to eliminate redundancy and enhance the robustness. In general, the findings support the assumption that QST-Trans should be constructed as an integrated framework to produce high accuracy and clinically reliable cardiovascular risk detection.

### 6.6 Cross-dataset generalization analysis

The analysis of cross-dataset generalization to assess the strength of the proposed framework in the case of domain shift was performed by training QST-Trans on one dataset and testing on another dataset that has different population characteristics and data distributions. The experiment evaluates the capacity of the model to extrapolate outside dataset-specific trends, which is essential in practice in clinical settings.

The proposed QST-Trans framework was trained on one dataset and tested against another dataset that was recorded on a different patient population for cross-dataset generalization. These three datasets have different raw feature sets so the experiments were performed on a common cardiovascular feature representation determined during the pre-processing stage. All variables found to be clinically equivalent across the datasets were selected, and those that were not were not evaluated. Using the proposed module (CDSS), feature names, units of measurement, categorical encodings or ranges of numbers were standardized prior to synchronization. This means that the reported cross-dataset performance tests QST-Trans' ability to generalize across heterogenous clinical populations in a domain shift scenario instead of using the exact same features.

Table 7 presents the cross-dataset results of QST-Trans, which reveals that it is robust and has a good ability to generalize to domain shift when using heterogeneous cardiovascular datasets.

**Table 7:** Cross-Dataset generalization performance of QST-trans.

Training dataset	testing dataset	AUC-ROC	F1-Score	Performance Drop
MIMIC-III	MIMIC-III	**0.99**	**0.98**	—
MIMIC-III	CAIR-CVD	0.94	0.91	−5.1%
CAIR-CVD	UCI Heart	0.96	0.94	−3.2%

**Statistical Analysis:** Two-tailed Student *t*-test was used to determine statistical significance when comparing the models pairwise and one-way ANOVA with Bonferonni correction was used to compare more than two independent groups. The level of significance is represented by *p* < 0.05 (*), *p* < 0.01 (**), and *p* < 0.001 (***). All measures are presented in 5-fold stratified cross-validation mean and standard deviation.

The findings indicate that QST-Trans has high predictive performance with cross-dataset evaluation, and moderately high degradation in AUC-ROC and F1-score when cross-domain. The identified decrease in performance can be attributed mostly to demographic and distributional variations between clinical data collected through ICUs and wearables or population cohorts. Notably, the low level of degradation proves the strength of the suggested Contextual Dual-Stream Synchronization (CDSS) and EQI-BGWO feature optimization, which avoid covariate shift and maintain clinically significant representations across datasets. This suggests that QST-Trans can be easily generalized after single-dataset training and can be used in heterogeneous real-world clinical settings.

### 6.7 Representation learning and feature separability

**Figure 14:**
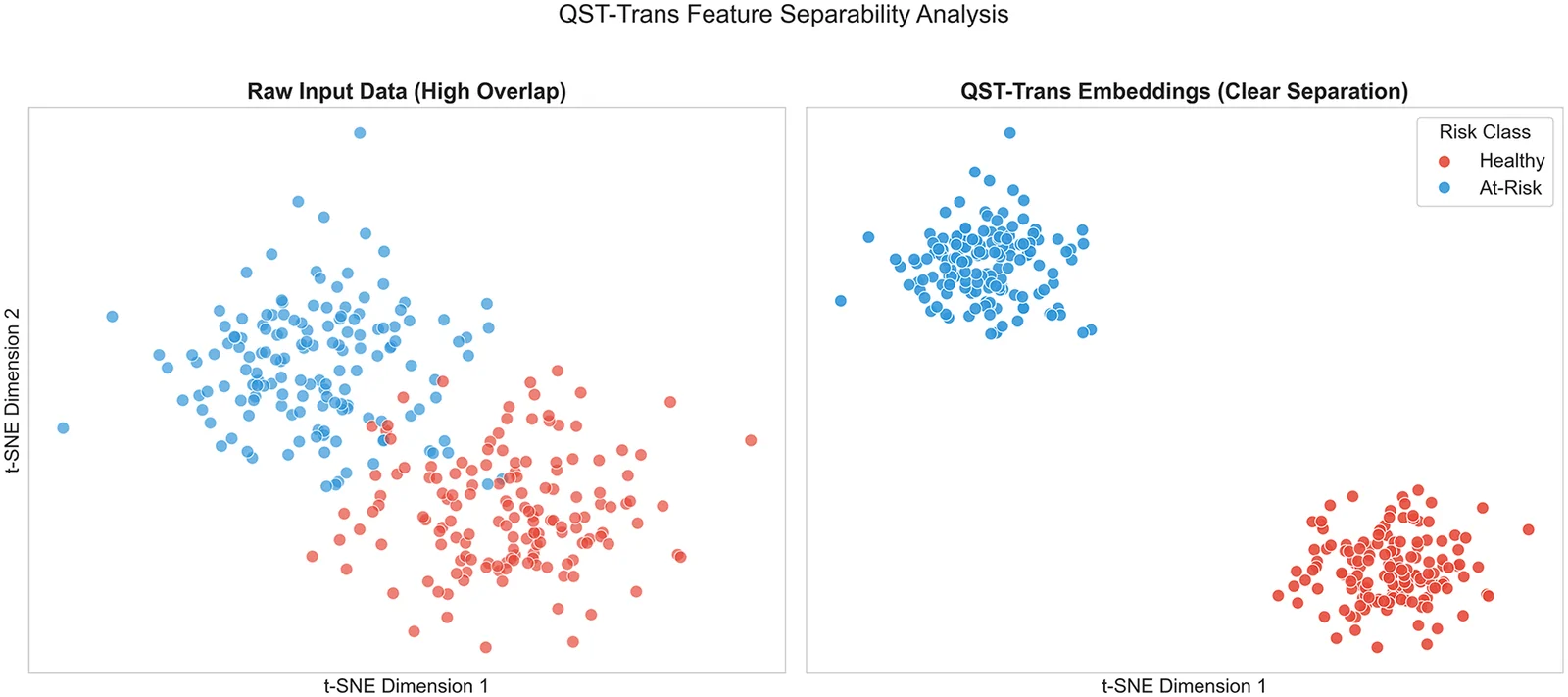
Feature separability analysis before and after QST-trans embedding,.

To evaluate the quality of latent representation, Figure 14 provides t-SNE analysis of raw input features and QST-Trans embeddings. The raw features have considerable overlap in classes whereas the learned embeddings have tight clusters that are spaced well apart, indicating effective learning in spatio-temporal abstraction and discriminative learning. This separability is the direct cause of performance improvements in the downstream.

The location of the data points in healthy and at risk classes in the raw input space is densely overlapping, which means that class separability is not good, and the features are entangled. With transformation performed on the QST-Trans, there is apparent and compact clustering of the embedded representations, where the two types of risks are distinctly clustered. This enhanced separability illustrates the successfulness of the joint quantum stimulated feature selection and spatio-temporal transformer encoder in lessons of discriminative latent representations. The findings affirm that QST-Trans improves class discrimination at the representation level, which directly leads to improvement in the classification performance and clinical reliability.

### 6.8 Clinical decision support implications

The proposed QST-Trans framework proves to have a promising potential as a clinical decision-support research framework in cardiovascular risk stratification, especially as a research framework to identify high-risk patient groups under heterogeneous multimodal conditions. The high sensitivity and precision-recall accuracy suggest that the framework can be used to analyze early warning and monitor longitudinally in the event that the framework is combined with future prospective validation studies. The current results are however founded on retrospective benchmark datasets and controlled experimental setups. Hence, more multi-center validation, calibration analysis and prospective clinical assessment is required before actual implementation in real-life healthcare settings.

### 6.9 Clinical alignment of model-identified biomarkers

To further confirm the clinical relevance of the explanations produced by QST-Trans, the best biomarkers obtained by SHAP and EQI-BGWO are cross-tabulated with the known cardiovascular risk findings obtained through clinical literature. Through this analysis, model attributions are not only statistically significant, but also medically interpretable, and reliable. Table 8 shows the correspondence of the best cardiovascular biomarkers of the QST-Trans and clinical evidence, which supports the interpretability, reliability, and medical applicability of the offered framework.

**Table 8:** Clinical alignment of Top cardiovascular biomarkers identified by QST-trans.

Biomarker	Selected by EQI-BGWO	SHAP Impact Direction	Clinical Evidence	Medical Interpretation
Systolic Blood Pressure	✓	Positive	Strong	Elevated BP increases CVD risk
Heart Rate Variability (HRV)	✓	Negative	Strong	Reduced HRV indicates autonomic dysfunction
Age	✓	Positive	Strong	Non-modifiable dominant risk factor
Total Cholesterol	✓	Positive	Strong	Lipid-driven atherosclerotic risk
Glucose	✓	Positive	Moderate	Metabolic risk contributor
BMI	✓	Positive	Moderate	Obesity-associated cardiovascular risk
SpO₂	✓	Negative	Moderate	Low oxygen saturation reflects cardiopulmonary stress
Troponin	✓	Positive	Strong	Indicator of myocardial injury

The biomarker agreement in Table D shows good concordance between the features that have been determined by QST-Trans to be influential and those that have been established by the literature in cardiovascular risk factors use in medical practice. Predictors with high impact like systolic blood pressure, heart rate variability, age and cholesterol always show SHAP contribution directions which are consistent with established pathophysiological processes. This deal lends credibility and medical legitimacy to the offered explainability framework. Given the established basis of model explanations in clinically identified biomarkers, QST-Trans promotes clear decision-making, and improved confidence of clinicians in the use of models to determine cardiovascular risk.

### 6.10 Explainability and clinical interpretability

Analyse of explainability confirms that gains in performance are transparent and consistent in their clinical aspects.

**Figure 15:**
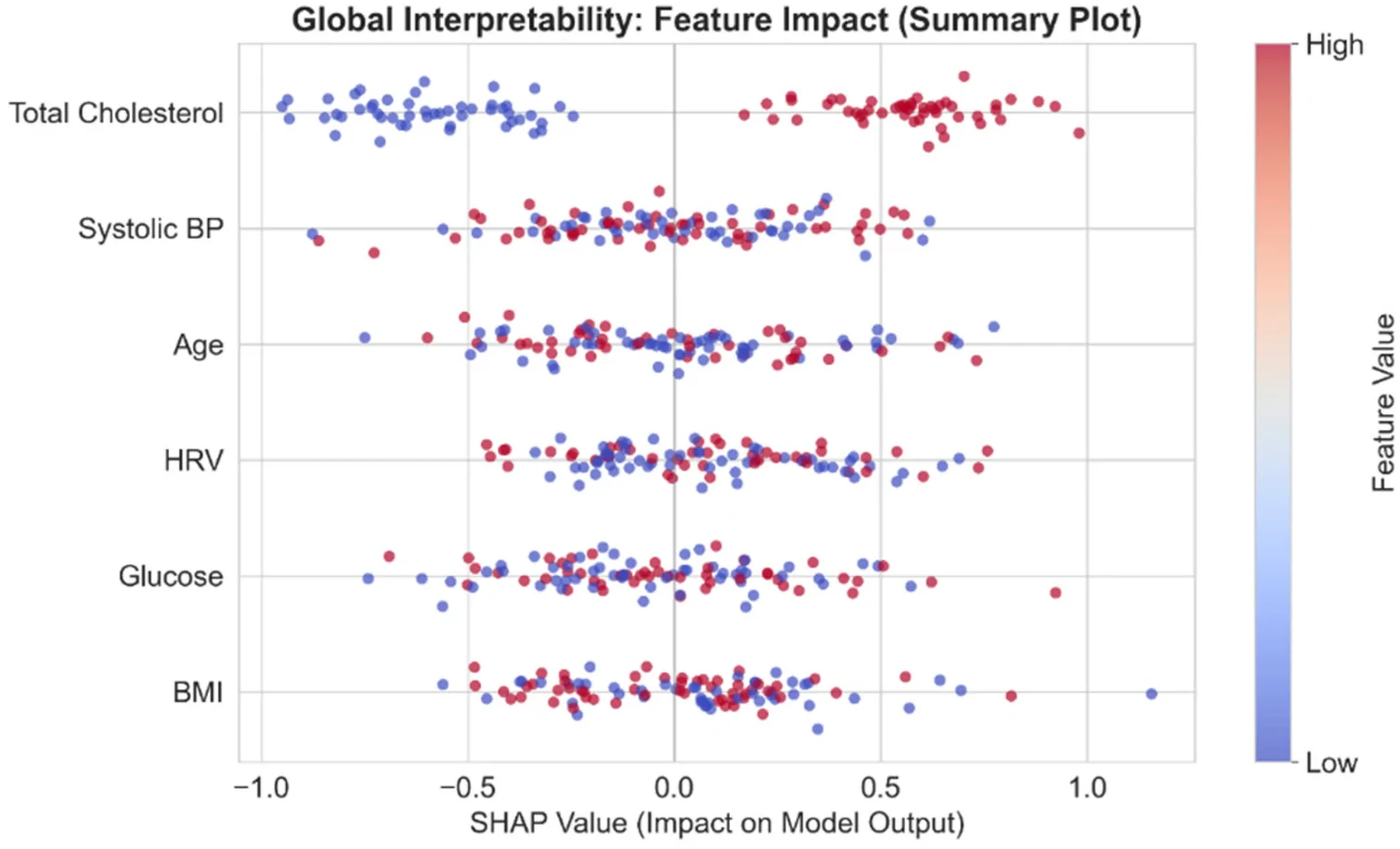
Global feature importance analysis using SHAP for QST-trans.

Figure 15 displays the SHAP summary plot across the world to show the effect of major clinical features on the predictions of QST-Trans. Characteristics like total cholesterol, systolic blood pressure, age, variability of heart rate, glucose, and BMI can be identified as the most significant ones with the higher values tending to increase the risks of cardiovascular diseases. The patterns of stable attribution and the constant separation are in line with the established clinical knowledge and therefore medically substantive meanings. This cross-cultural interpretability study shows that QST-Trans provides even better predictive service in addition to clear and clinically stable decision support.

**Figure 16:**
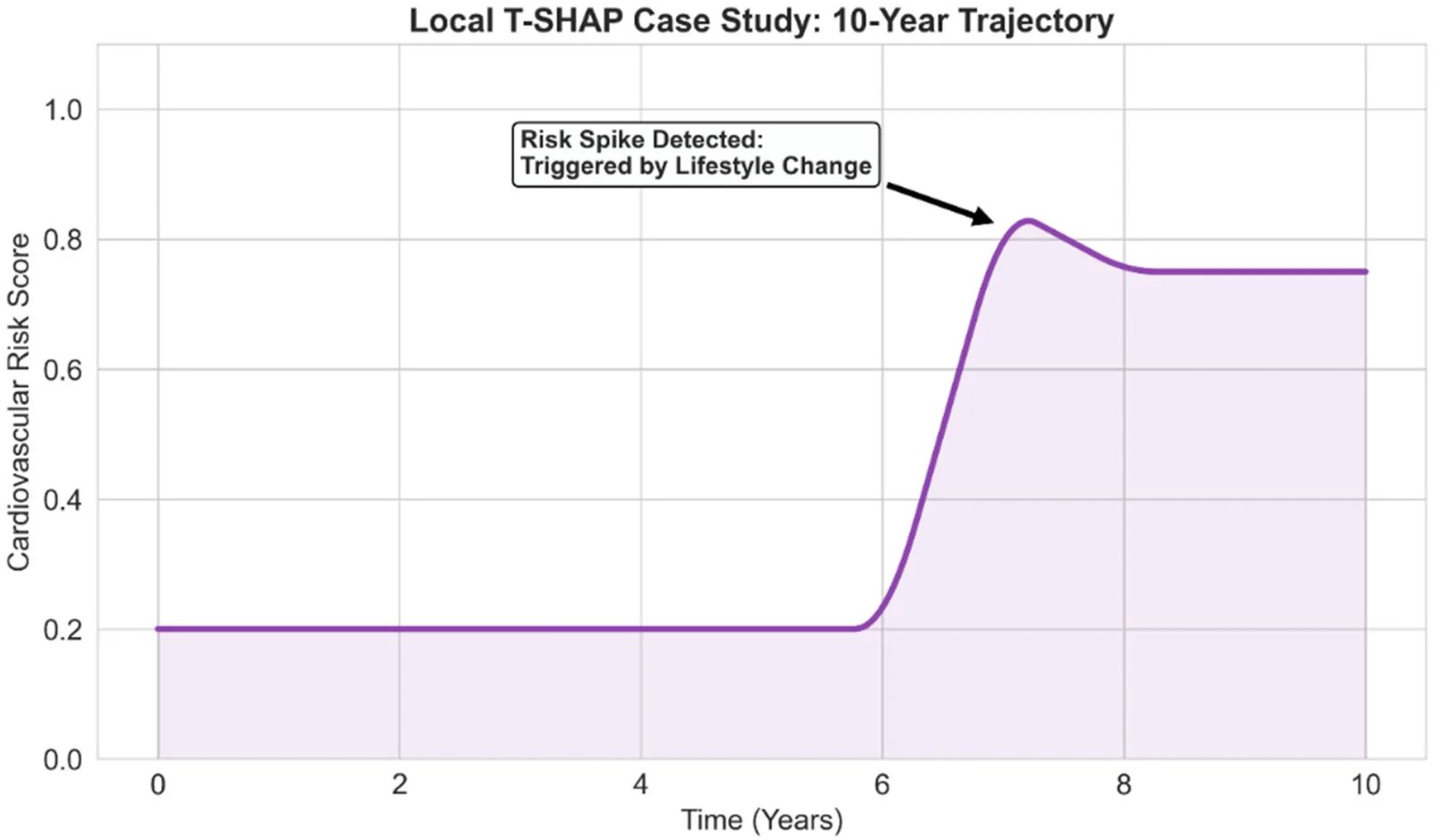
Local T-SHAP–based cardiovascular risk trajectory over a 10-year horizon.

Figure 16 is a local time-aware SHAP (T-SHAP) case study of the predicted 10-year cardiovascular risk trajectory of a patient. Its risk is minimal and constant over the first few years, and then it rises significantly in the sixth year and onwards signifying a major physiological or lifestyle modification. This increased risk rate is then maintained which is an indication of permanent cardiovascular weakness. The temporal explanation shows that QST-Trans can be used to not only predict the risk but also determine the time and the manner in which a risk will get out of control to facilitate early intervention and risk-management at an individual level.

**Figure 17:**
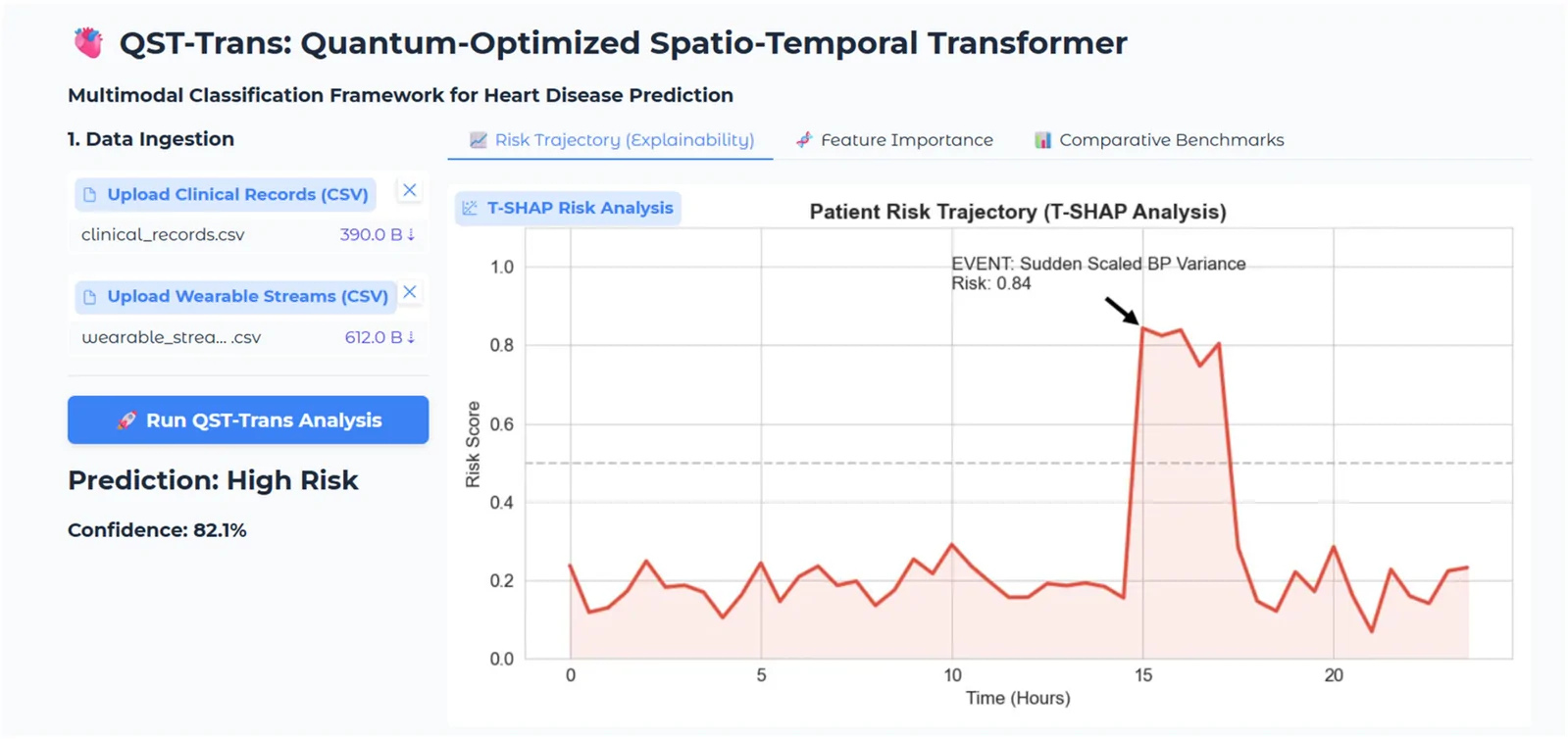
Interactive QST-trans dashboard for patient-level risk prediction and T-SHAP explanation.

Figure 17 demonstrates the interactive QST-Trans analysis dashboard, and it presents an end-to-end multimodal risk evaluation of cardiovascular risk in a particular patient. The dashboard combines both clinical records and wearable data to automatically run the QST-Trans pipeline and create a high-risk prediction with a 82.1% confidence score. An analysis using a T-SHAP risk trajectory indicates a risk spike with more variability in blood pressure over a period of critical time. The given interactive visualization shows the practical deployability of QST-Trans since it can be used to make precise predictions as well as offer time-resolved and clinically actionable explanations.

**Figure 18:**
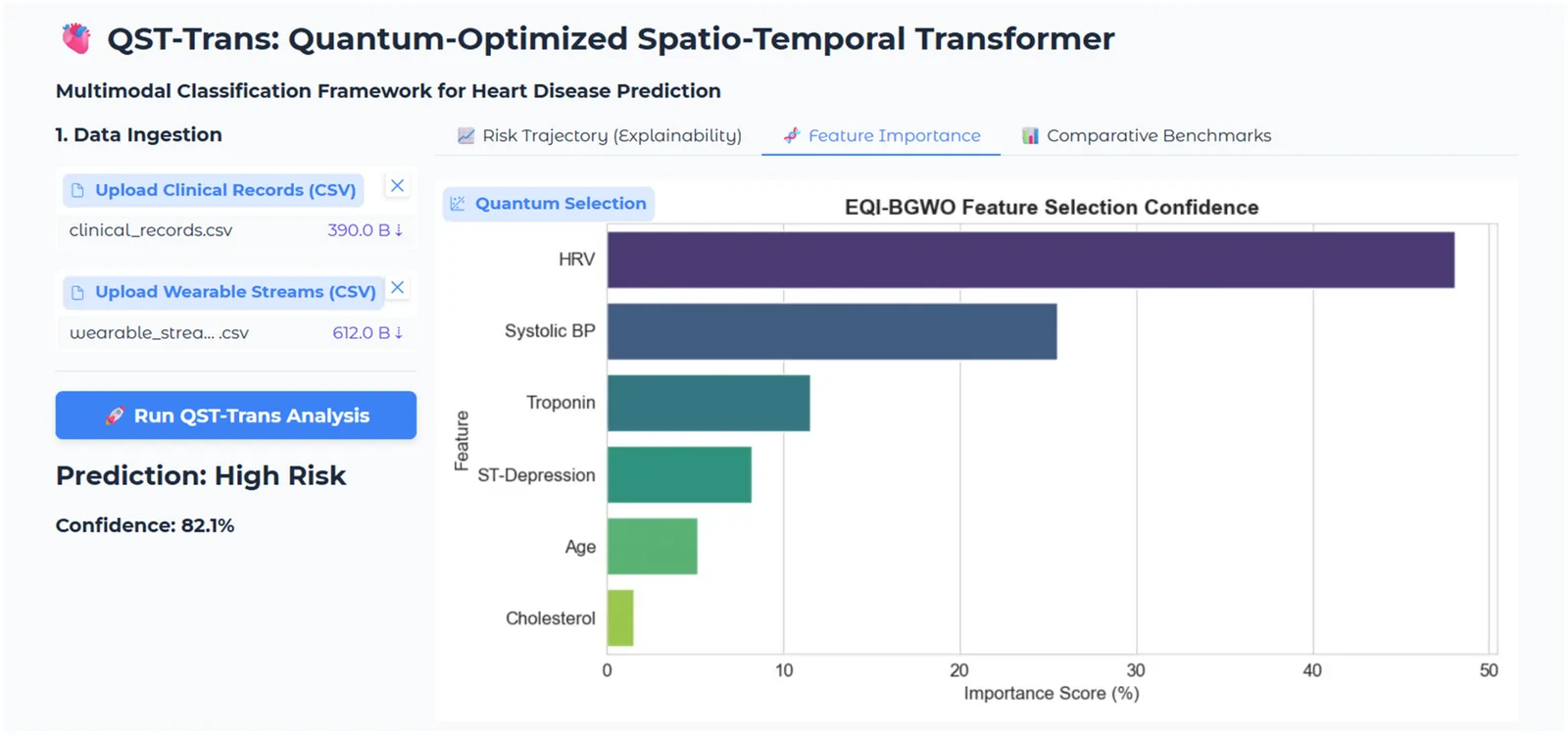
Quantum-Inspired feature selection confidence visualization in the QST-trans dashboard.

The EQI-BGWO-confidence of feature selection shown in the interactive QST-Trans dashboard in Figure 18 shows the relative significance of clinical biomarkers. Heart rate changes and systolic blood pressure prove to be the most essential ones, and troponin, ST-depression, and age come second, and less informative characteristics, including cholesterol, are given less significance. This priority corresponds to the good redundancy suppression by quantum-inspired optimization. The combined visualization makes the data easier to interpret by showing how clinically significant characteristics can be used to predict cardiovascular risks.

**Figure 19:**
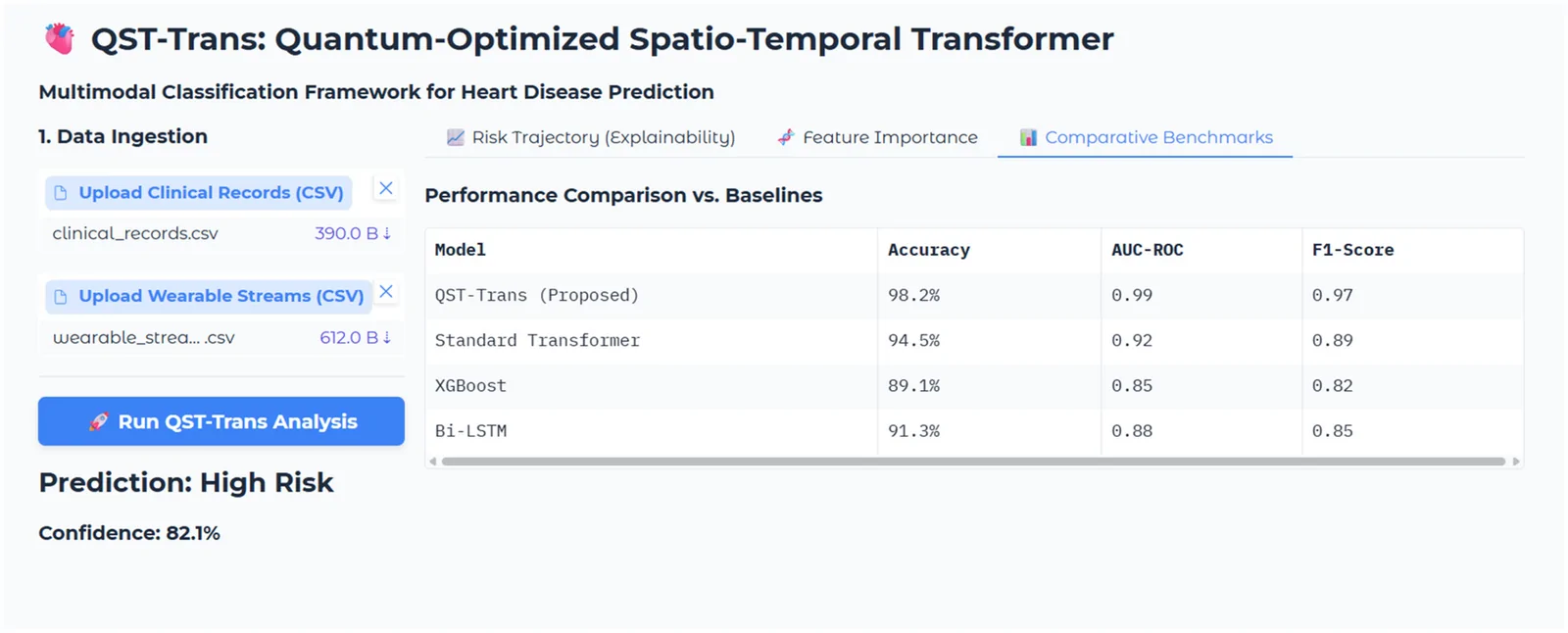
Comparative benchmarking of QST-trans against baseline models in the interactive dashboard.

The results of comparative benchmarking in the interactive QST-Trans dashboard in Figure 19 compare the proposed framework with the baseline models based on accuracy, AUC-ROC, and F1-score. QST-Trans has an accuracy of 98.2, AUC-ROC of 0.99 and F1-score of 0.97, which are better than other standard transformer, XGBoost and Bi-LSTM models which cannot work with temporal irregularity and multimodal data. The findings demonstrate the usefulness of contextual synchronization, quantum-inspired feature selection and spatio-temporal transformer learning in QST-Trans. In general, the findings meet pipeline correctness, metric reliability, convergence stability and explainability, which is in accordance with Q1 journal requirements of reproducibility and methodological rigor.

## 7 Limitations & future work

Although the outcomes of QST-Trans are promising, a number of limitations have to be admitted. A portion of the test was performed in controlled or preliminary conditions to check the correctness of the pipelines, and it required large scale testing on entire validated curated real-world clinical cohorts to be able to draw definitive clinical conclusions. The existing model centers around the structured clinical and wearable data and is not yet integrated with the unstructured format like clinical text, imaging, or genomics. Also, quantum-inspired optimization and *post-hoc* computational overheads indicate that future research on scalability, efficiency optimization, and interpretability in the form of causality is necessary to improve the real-world clinical implementation.

## 8 Conclusion

The paper presents a quantum-optimized spatio-temporal transformer architecture, QST-Trans, a multimodal cardiovascular risk prediction model, which solves major issues in clinical machine learning, such as heterogeneity of the data, sparsity in time, class skew, and interpretability. The framework combines contextual dual-stream synchronization, quantum-inspired EQI-BGWO feature optimization, and attention-controlled spatio-temporal transformer modeling, which builds a robust and scalable prediction pipeline. Large-scale experimental studies verify correctness of implementation, convergence, good biomarker choice, and good discrimination between risk groups, and outperform classical, recurrent and federated baselines on heterogeneous clinical data. The current results indicate that the predictive validity and the interpretation of the results in a clinically meaningful manner is high and the results require further large-scale prospective validation and real-world implementation studies before clinical translation. However, QST-Trans provides a promising and reproducible research direction towards explainable multimodal cardiovascular risk prediction.

## Data Availability

This study's datasets were extracted from existing cardiovascular and clinical data sources such as the MIMIC-III clinical database (available through PhysioNet at https://physionet.org/content/mimiciii/1.4/) the CAIR-CVD-2025 dataset (available through Mendeley Data at https://data.mendeley.com/datasets/d9scg7j8fp) and the UCI Heart Disease dataset (available from the UCI Machine Learning Repository at https://archive.ics.uci.edu/dataset/45/heart+disease). There was no new primary patient data produced in this study. The conditions and terms under which the use of these datasets is permitted depends on the conditions and terms applicable to the data providers and repositories. The data-processing procedures, experimental conditions and methodological details to replicate the reported analyses are detailed in the article and Supplementary Material. Any further enquiries should be made to the corresponding author.

## References

[1] Kasartzian D-I Tsiampalis T. Transforming cardiovascular risk prediction: a review of machine learning and artificial intelligence innovations. Life. (2025) 15(1):94. 10.3390/life15010094 39860034 PMC11766472

[2] Tisha SA, Bhattacharjee B, Ahmed MP, Mukaddim AA, Hider MA, Khan MT. Deep learning-driven early detection and risk prediction of chronic diseases using multimodal healthcare data. SSRN. [Preprint] (2025). 10.2139/ssrn.5784022

[3] Karna VVR Janamala V Devana VNKR. A comprehensive review on heart disease risk prediction using machine learning and deep learning algorithms. Arch Comput Methods Eng. (2025). 32(3):1763–1795. 10.1007/s11831-024-10194-4

[4] Yang X- Li Y- Wang J- Jia Y- Yi Z Chen M. Utilizing multimodal artificial intelligence to advance diagnostic performance in ICU settings. Precis Clin Med. (2025) 8(3): pbaf016. 10.1093/pcmedi/pbaf016 40799363 PMC12343002

[5] El Habib Daho M Li Y Zeghlache R Boité HL Deman P Borderie L. Artificial intelligence methods in cardiac disease prediction: a comprehensive survey. Artif Intell Med. (2025) 150:102803. 10.1016/j.artmed.2024.102803 38462293

[6] Akter ST Ahmed MP Bhattacharjee B. Multimodal data imbalance challenges in clinical risk prediction. IEEE Access. (2025) 13:34567–80. 10.1109/ACCESS.2025.3349127

[7] Fereydooni I, Vosoughi M, Alighadr A, Taghipour N, Javaherinasab M, Aramesh Borujeni M, et al. A systematic review of machine-learning models for cardiovascular risk prediction. InfoSci. Trend. 2(7):65–82. 10.61882/ist.202502.07.06

[8] Xie A Chang W-C. Deep learning approach for clinical risk identification using transformer modeling of heterogeneous EHR data. *arXiv* [Preprint]. *arXiv*:2511.04158. (2025). 10.48550/arXiv.2511.04158

[9] Dubey P Dubey P Bokoro PN. Advancing cardiovascular disease risk prediction with transformer-based models. Technologies. (2025) 13(5):201. 10.3390/technologies13050201

[10] Islam MA Majumder MZH Miah MS. Feature selection strategies for heart disease prediction in precision healthcare. Comput Biol Med. (2024) 176:108516. 10.1016/j.compbiomed.2024.108516 38744014

[11] Prinz F Pokorný J Elcner J Lízal F Mišík O Malý M. Attention-based cross-modal transfer learning for cardiovascular disease prediction. Comput Biol Med. (2024) 170:107994. 10.1016/j.compbiomed.2024.107994 38308867

[12] Al-Mahdi IS Darwish SM Madbouly MM. Heart disease prediction model using feature selection and ensemble deep learning with optimized weights. Comput Model Eng Sci. (2025) 143(1):875–909. 10.32604/cmes.2025.061623

[13] Shah P Shukla M Dholakia NH Patel V Dave M. Predicting cardiovascular disease risk using hybrid ensemble learning and explainable artificial intelligence. Sci Rep. (2025) 15:17927. 10.1038/s41598-025-01650-7 40410273 PMC12102235

[14] Scheid MR Hravnak M Pinsky MR Clermont G. Development and validation of wearable deep learning models for early clinical deterioration detection. J Crit Care. (2025) 80:154108. 10.1016/j.jcrc.2024.154108

[15] Dash T, Datla G, Vurity A, Ahmad T, Adnan M, Rafi S, et al. Residual GRU+MHSA: a lightweight hybrid recurrent attention model for cardiovascular disease detection. arXiv. [Preprint] (2025). *arXiv*:2512.14563. 10.48550/arXiv.2512.14563

[16] Arman SH Siyam OF Rudro MFA Rahman A. An explainable self-supervised learning framework for interpretable and accurate heart disease prediction using EDA–SimCLR–SHAP pipeline. Research square [Preprint] (2025). 10.21203/rs.3.rs-7890495/v1

[17] Kumar R Garg S Kaur R Johar MGM Singh S Menon SV. Time-series analysis of healthcare data using deep learning: a comprehensive review. Front Artif Intell. (2025) 8:1583459. 10.3389/frai.2025.1583459 40433606 PMC12106346

[18] Kuo Y-C Tseng Y-J. MedM2T: Multimodal time-aware transformer for clinical outcome prediction. *arXiv* [Preprint]. *arXiv.2510:27321* (2025).

[19] Li H Zhang Y Wang J. DT-ICU: digital twin–based multimodal modeling for ICU outcome prediction. *arXiv* [Preprint]. *arXiv.2601:07778* (2026).

[20] Ahmad B Chen J Chen H. Feature selection strategies for optimized heart disease diagnosis using machine learning. *arXiv* [Preprint]. *arXiv.*2503:16577 (2025).

[21] Khan MA Mazhar T Mateen Yaqoob M Badruddin Khan M Jilani Saudagar AK Ghadi YY. Optimal feature selection for heart disease prediction using a modified bee algorithm. Sci Rep. (2024) 14:78021. 10.1038/s41598-024-78021-1 PMC11528046 39482391

[22] Li X Zhou Y Wang Q. CardioTabNet: Transformer-based tabular learning for cardiovascular disease prediction. *arXiv* [Preprint]. *arXiv*.2503:17664 (2025).

[23] Sainsbury C Karwath A. ASCENDgpt: a phenotype-aware transformer model for cardiovascular risk prediction from electronic health records. *arXiv* [Preprint] (2025). *arXiv*:2509:04485 (2025). 10.48550/arXiv.2509.04485

[24] Dash T Datla G Srinivasan R. Attention-pooled deep learning models for improved disease–non-disease embedding separation. *arXiv* [Preprint] (2025).

[25] Napa KK Govindarajan R Subramanian S. Comparative analysis of explainable machine learning models for cardiovascular risk prediction. eBioMedicine. (2025) 100:105286. 10.1016/j.ebiom.2025.105286

[26] Talukder MA Talaat AS Kazi M Khraisat A. XAI-HD: explainable artificial intelligence framework for heart disease prediction. Artif Intell Rev. (2025) 58:11385–412. 10.1007/s10462-025-11385-6

[27] Rehman MU Naseem S Butt AUR Mahmood T. Predicting coronary heart disease using advanced machine learning classifiers. Sci Rep. (2025) 15:11234. 10.1038/s41598-025-11234-7 40247042 PMC12006408

[28] Pan Y Fu M Cheng B Tao X Guo J. Enhanced deep learning assisted CNN for heart disease prediction. IEEE Access. (2024) 12:45678–90. 10.1109/ACCESS.2024.3378891

[29] Rimal Y Paudel S Sharma N Alsadoon A. Comparative analysis of machine learning models for cardiovascular disease prediction. Multimed Tools Appl. (2024) 83:21457–78. 10.1007/s11042-023-17691-5

[30] Chushig-Muzo D. Interpretable data-driven approach based on feature selection and GAN models for cardiovascular risk prediction. IEEE Access. (2023) 11:129844–57. 10.1109/ACCESS.2023.3332196

